# Targeted sequencing of DNA/RNA combined with radiomics predicts lymph node metastasis of papillary thyroid carcinoma

**DOI:** 10.1186/s40644-024-00719-2

**Published:** 2024-06-17

**Authors:** Runjiao Zhang, Linfei Hu, Yanan Cheng, Luchen Chang, Li Dong, Lei Han, Wenwen Yu, Rui Zhang, Pengpeng Liu, Xi Wei, Jinpu Yu

**Affiliations:** 1 Cancer Molecular Diagnostics Core, Key Laboratory of Cancer Immunology and Biotherapy, Key Laboratory of Cancer Prevention and Therapy, Tianjin Medical University Cancer Institute and Hospital, National Clinical Research Center of Cancer, Tianjin, China; 2 Tianjin’s Clinical Research Center for Cancer, Tianjin, China; 3 Department of Thyroid and Neck Tumor, Key Laboratory of Cancer Prevention and Therapy, Tianjin Medical University Cancer Institute and Hospital, National Clinical Research Center for Cancer, Tianjin, China; 4 Department of Diagnostic and Therapeutic Ultrasonography, Key Laboratory of Cancer Prevention and Therapy, Tianjin Medical University Cancer Institute and Hospital, National Clinical Research Center of Cancer, Tianjin, China; 5 Department of Immunology, Key Laboratory of Cancer Immunology and Biotherapy, Tianjin Medical University Cancer Institute & Hospital, National Clinical Research Center for Cancer, Tianjin’s Clinical Research Center for Cancer, Tianjin, China

**Keywords:** DNA damage repair, Mutation, Next generation sequencing, Thyroid carcinoma, Ultrasonography, Predictive model, Clinical application

## Abstract

**Objective:**

The aim of our study is to find a better way to identify a group of papillary thyroid carcinoma (PTC) with more aggressive behaviors and to provide a prediction model for lymph node metastasis to assist in clinic practice.

**Methods:**

Targeted sequencing of DNA/RNA was used to detect genetic alterations. Gene expression level was measured by quantitative real-time PCR, western blotting or immunohistochemistry. CCK8, transwell assay and flow cytometry were used to investigate the effects of concomitant gene alterations in PTC. LASSO-logistics regression algorithm was used to construct a nomogram model integrating radiomic features, mutated genes and clinical characteristics.

**Results:**

172 high-risk variants and 7 fusion types were detected. The mutation frequencies in *BRAF*, *TERT*, *RET*, *ATM* and *GGT1* were significantly higher in cancer tissues than benign nodules. Gene fusions were detected in 16 samples (2 at the DNA level and 14 at the RNA level). *ATM* mutation (*ATM*^MUT^) was frequently accompanied by *BRAF*^MUT^, *TERT*^MUT^ or gene fusions. *ATM*^MUT^ alone or *ATM* co-mutations were significantly positively correlated with lymph node metastasis. Accordingly, *ATM* knock-down PTC cells bearing *BRAF*^V600E^, *KRAS*^G12R^ or *CCDC6-RET* had higher proliferative ability and more aggressive potency than cells without *ATM* knock-down in vitro. Furthermore, combining gene alterations and clinical features significantly improved the predictive efficacy for lymph node metastasis of radiomic features, from 71.5 to 87.0%.

**Conclusions:**

Targeted sequencing of comprehensive genetic alterations in PTC has high prognostic value. These alterations, in combination with clinical and radiomic features, may aid in predicting invasive PTC with higher accuracy.

**Supplementary Information:**

The online version contains supplementary material available at 10.1186/s40644-024-00719-2.

## Introduction

Papillary thyroid carcinoma (PTC) is the dominant sub-type of thyroid carcinoma (accounting for 70–80%) [1–4]. The 10-year overall survival rate of patients with PTC exceeds 95%. Some cases are characterized by small primary tumors and extensive lymph node metastases, which significantly induce unfavorable relapse and poor clinical outcomes. Thyroid carcinoma shows highly heterogeneous biological characteristics during occurrence and development. Driver gene alterations are important molecular events fueling the growth and metastasis of PTC, among which driver gene mutations and fusions are most common. These alterations play crucial roles in maintaining the genetic and phenotypic heterogeneity of cancer. Mutated driver genes are increasingly being identified in PTC; however, single driver gene variation cannot comprehensively explain the heterogeneity observed in PTC development and progression. Therefore, a feasible method to detect multiple-gene signatures is essential to predict invasive PTC phenotypes, and provide optimal and efficient therapeutic regimens.

Next generation sequencing (NGS) is playing an increasingly important role in the detection of gene alterations. In contrast to Sanger sequencing, NGS uses microarrays to read and sequence millions of short sequences simultaneously. NGS can also detect DNA in tested samples at relatively low cost. Owing to the limitations of DNA sequencing in detecting gene fusions, the detection of mutation and fusion simultaneously by DNA/RNA dual sequencing has become a new research focus [5–8]. Currently, most studies applying NGS technology have been aimed at differentiating benign and malignant thyroid nodules, to aid in early diagnosis and clinical management of patients with indeterminate thyroid nodules [5–11]. However, limited NGS-associated biomarker studies have focused on PTC prognosis because driver gene variations alone cannot predict invasive PTC phenotypes with high accuracy and reliability [12]. Therefore, investigating whether NGS-based targeted sequencing technology of DNA/RNA might help establish a multiple-gene signature to predict invasive PTC phenotypes in clinical settings should prove valuable.

Conventional preoperative ultrasound is an easy, safe and non-invasive procedure routinely used to predict lymph node metastasis in patients with PTC in clinical settings [13]. Radiomic features are established on the basis of ultrasonographic images, and can be computed from grayscale images to reflect the texture and morphology of tumors [14–17]. And radiomics has been applied to evaluate various tumors, such as hepatocellular carcinoma, breast cancer, thyroid carcinoma and renal tumors [18–21]. As radiomics has attracted attention because of its precise diagnosis, radiomics-based studies for assessing lymph node metastasis risk in patients with thyroid carcinoma have been proposed in recent years [22]. However, few studies have predicted lymph node metastasis of thyroid carcinoma on the basis of a combination of gene alterations, clinical features and radiomics.

In this study, we explored PTC heterogeneity by comparing mutations and fusions between cancer tissues and benign nodules through NGS-based targeted sequencing technology of DNA/RNA. Furthermore, we conducted experiments to explore the pro-tumoral effects of concomitant gene alterations in PTC in vitro. Lymph node metastasis has an impact on recurrence and the choice of surgical methods, and ultimately affects the quality of patients’ life [23]. Thus, we aimed to propose a model for predicting lymph node metastasis integrating gene alterations, clinical features and radiomic features, and then to provide evidence to support accurate diagnosis and treatment of thyroid carcinoma.

## Materials and methods

### Patient information

Thyroid nodule tissues from 182 cases were obtained from the Department of Thyroid and Neck Tumors and the Department of Diagnostic and Therapeutic Ultrasonography of Tianjin Medical University Cancer Institute and Hospital, Tianjin, China. Samples obtained by fine-needle aspiration (FNA) were collected from Chinese patients aged 21–72 years between December 2016 and May 2018. Detailed including/excluding criteria was shown as below:

Inclusion: patients with suspected malignant thyroid nodules diagnosed by ultrasound.

Exclusion: (1) patients with severe bleeding tendency or coagulation disorders; (2) patients allergic to anesthetics; (3) nodules close to large blood vessels or vital nerves; (4) patients with a propensity for severe puncture complications.

Thyroid Imaging Reporting and Data System (TI-RADS) has been used in numerous centers in China and has been standardized to improve PTC diagnosis. A total of 182 thyroid nodule samples were graded according to ultrasound, among which TI-RADS 2 (*n* = 1) and 3 (*n* = 5) were classified as benign, and TI-RADS 5 (*n* = 25) and 6 (*n* = 3) were classified as malignant [24]. As TI-RADS 4 was suspected to be malignant, samples of TI-RADS 4 were further classified as benign (*n* = 52) and malignant (*n* = 96) by combining fine-needle aspiration cytology. 124 cases of cancer tissues underwent surgery subsequently, and were verified by pathological examination (Table S1, Table S2). All sample categorization was confirmed in accordance with the guidelines for diagnosis and treatment of thyroid nodules and differentiated thyroid carcinoma (2023). Among the 124 PTC cases, the nuclei acids of 81 matched para-carcinoma tissues were also prepared and sequenced all together.

### Panel-based genomic sequencing and filtering of variants

A total of 182 DNA samples and 182 RNA samples were extracted from FNA tissues, and the purity, on the basis of OD_260/280_ ratio, was 1.8–2.0. Genetic mutations in 49 hotspot genes in thyroid carcinogenesis, including 13 proto-oncogenes, seven tumor suppressor genes and 29 genetic susceptibility genes, were detected with DNA target sequencing, and all detected sites were coding regions. Furthermore, 36 types of gene fusion frequently reported in thyroid carcinoma were detected with RNA target sequencing (Table S3) on the Illumina MiSeq platform (Illumina, San Diego, CA, USA), with an average sequencing depth of 3353×.

Variants were excluded when any of the following criteria were met:

- Variants had low allele frequency (< 1%).
- Variants were detected with a low allele depth (AD), AD[0] + AD[1] < 50.
- The FS (Phred-scaled p-value with Fisher’s exact test to detect strand bias) was > 60.
- The symmetric odds ratio of the 2 × 2 contingency table to detect strand bias was > 3.
- The ECNT (number of events in this haplotype) was > 10.

### Genetic alteration analysis

FastQC and Trimmomatic were used to evaluate the quality of sequencing data. The sequencing data were aligned to the human reference genome (GRCh37/Hg19). Sentieon and Starfusion software were used to obtain detailed SNP indel and fusion information, respectively. The vcf results of SNP indels were used for annotation with information from databases (including ClinVar, COSMIC, DBNSFP, DBSCSNV, DBSNP, ESP6500, EXAC, G1000, GNOMAD, HGMD, ICGC and REFGENE) in the 2019 version of Annovar tools and comments library, to obtain SNP indel annotation results.

Information on mutations, including mutation sites, base substitutions and amino acid replacements, was extracted from the original data. The pathogenic loci were screened using ClinVar database and predictive tools - InterVar, FATHMM and Polyphen2.

### Functional enrichment analysis of mutated genes in cancer tissues

Enrichment analysis with the Reactome pathway database was performed for 31 mutated genes with the online functional enrichment tool KOBAS 3.0 (KOBAS: http://kobas.cbi.pku.edu.cn/kobas3/?t=1). All signal transduction pathways were further investigated. The pathways were sorted according to the number of foreground genes, and the top 11 pathways were selected for display.

### Image segmentation, preprocessing and extraction of radiomic features

The ultrasound images of the primary thyroid lesions of 108 PTC samples were retrieved from the picture archiving and communication system (PACS) at Tianjin Medical University Cancer Institute and Hospital (Figure S1). Thyroid nodules on the ultrasound images were contoured by one junior radiologist and confirmed by a senior radiologist (Dr. X. Wei, with 15 years of experience in thyroid carcinoma ultrasound diagnosis).

Radiomic feature extraction was performed in MATLAB 2018a (MathWorks Inc., Natick, MA, USA). A set of 854 radiomic features were extracted from each of the segmented objects for each patient with PTC with existing automated computer program - PyRadiomics package. These features were divided primarily into four groups: (1) morphological features, such as volume, maximum diameter and maximum probability; (2) first-order statistics; (3) second-order statistics or textural features, including gray-level co-occurrence matrix, gray-level run-length matrix and gray-level size zone matrix; and (4) wavelet feature [25]. Z-score normalization was performed as a preprocessing step to ensure the repeatability of the results.

### Development of the multi-feature integration nomogram model for lymph node metastasis prediction

The patients were randomly divided into a training set (*n* = 76) and a test set (*n* = 32) at a ratio of 7:3. Data in the training set were used to develop the radiomics signature model for predicting lymph node metastasis of PTC. To minimize overfitting and decrease the bias from 854 radiomic features in the model, the least absolute shrinkage and selection operator (LASSO) algorithm was used to select the best features precisely. Then the error was estimated through cross-validation, and the lambda value that minimized cross-validation error was determined. Collinearity of the features was evaluated by variance inflation factor (VIF). Three most important features with non-zero coefficients were finally selected, including wavelet-LHL_glrlm_Short Run Emphasis, wavelet-HLH_glszm_Size Zone Non-Uniformity and wavelet-HLH_glszm_Small Area Low Gray Level Emphasis. And a Radscore was calculated for each patient based on the features selected (Radscore = -0.4662*wavelet-LHL_glrlm_ Short Run Emphasis + 0.28125*wavelet-HLH_glszm_Size Zone Non-Uniformity + 0.00306* wavelet- HLH_glszm_Small Area Low Gray Level Emphasis). Meanwhile, a radiomics signature model was developed through binary logistic regression analysis. Further, mutated genes associated with lymph node metastasis in the univariate analysis, including *RET, ATM, PIK3CA, TERT, GGT1* and fusions, were incorporated to construct a gene signature model. And a clinical feature model based on clinical characteristics associated with PTC (including age, gender, extrathyroidal extension, Hashimoto’s thyroiditis, tumor stage, family history and tumor size) was also constructed. Furthermore, a multi-feature integration nomogram model predicting lymph node metastasis of PTC were constructed integrating mutated genes, clinical characteristics and radiomic features (Figure S2).

### Cell line construction

The human thyroid cancer cell lines B-CPAP (BRAF p.V600E), Cal-62 (KRAS p.G12R) and TPC-1 (CCDC6-RET), and the normal thyroid epithelial cell line Nthy-ori-3-1, were purchased from Bestbay and validated through short tandem repeat DNA profiling and mycoplasma testing. All cell lines were maintained in a 37℃, 5% CO_2_ humidified chamber in 10% FBS (Fetal Bovine Serum) + RPMI 1640/Dulbecco’s modified Eagle’s medium (Gibco BRL).

### siRNA transfection

ATM siRNA and the non-targeting (NC) siRNA were synthesized by HanBio (Shanghai, China). A 3 µL volume of siRNA or NC in Lipofectamine 2000 reagent (Biosky, Nanjing, China) was transfected into B-CPAP, Cal-62, TPC-1 and Nthy-ori-3-1 cells according to the manufacturer’s instructions. After 48 h, the transfected cells were harvested for validation and sequential experiments.

### Quantitative real-time PCR analysis

Total RNA was extracted from cells with a TRIzol kit according to the manufacturer’s protocol. Quantitative real-time PCR was performed with a TB Green Premix Ex Taq Kit (Takara), according to the manufacturer’s instructions. Data were collected and analyzed with a LightCycler 480 instrument (Roche). β-actin was used as an internal control for mRNA expression. Primers are listed in Table S4. Relative mRNA levels in each sample were calculated on the basis of their Ct values normalized to the Ct value of the internal control, with the formula 2^−ΔCt^ (ΔCt = Ct_target gene_ − Ct_β−actin_).

### Western blotting

Western blotting was performed to detect levels of ATM (1:1000, Abcam, #ab32420) and GAPDH (1:3000, Santa Cruz, #sc-32233) proteins in thyroid cancer cells. Protein detection was performed with a ChemiDoc XRS Detection System (Bio-Rad). The relative densities of protein bands were determined by comparison with the band densities of proteins of interest with those of the internal reference GAPDH.

### Annexin V assays

Annexin V assays were used to detect apoptosis of cancer cells. Cells were stained with FITC-Annexin V and propidium iodide from an Apoptosis Detection Kit (BD Biosciences). Cells with positive expression of Annexin V and negative expression of propidium iodide were early apoptotic cancer cells.

### Cell counting Kit-8 (CCK8) assays

Cells (2 × 10^3^ cells/well) were seeded into 96-well plates in a 100 µL volume. CCK8 solution (10 µL; US EVERBRIGHT INC, Suzhou, China) was added to each well for different time periods (0, 24, 48, 72 or 96 h), then incubated for 2 h at 37℃. Cell viability was determined according to the absorbance at 450 nm.

### Invasion assays

Cells (3 × 10^4^ cells/well) were suspended in 200 µL of serum-free RPMI-1640/ Dulbecco’s modified Eagle’s medium and seeded into the top wells of a transwell apparatus, which were coated with 50 mg/L Matrigel (1:7 diluted solution; BD Biosciences) and air dried at 37℃. The lower wells contained 500–600 µL complete medium with 10% FBS in a 24-well plate outside the chambers. Culturing was performed in a 37℃, 5% CO_2_ incubator. All of the cells in chambers with Matrigel were collected after incubation for 48 h. Subsequently, chambers with filtered cells were fixed with methanol and stained with 1% crystal violet. Cells that did not cross the membrane were removed. The stained cells from five selected views were observed under a light microscope at 200× magnification.

### Immunohistochemistry assays

Paraffin-embedded tissue slides were heated for 1–2 h at 65°C, deparaffinized in xylene and rehydrated through a graded alcohol series. Antigens were retrieved by heating in EDTA antigen repair solution (pH 9.0) for 20 min. Endogenous peroxidase activity was quenched in a hydrogen peroxide bath for 30 min. Subsequently, the slides were incubated overnight at 4°C with rabbit monoclonal antibody to ATM (1:100, Abcam, #ab32420), which was validated in other tissues [26, 27]. And the positive staining was localized to nucleus. ATM positively stained cells were counted in five fields at 200× magnification, and the average value of the cells in the five fields was calculated as positive cell counts.

### Statistical analysis

Statistical analysis was performed using SPSS 25.0 (SPSS, Inc., Chicago, IL, USA), R language (version 4.1.2) and GraphPad Prism 8.0.1 software (GraphPad, Inc., La Jolla, CA, USA). All presented data are from at least three independent biological experiments and are shown as mean ± standard deviation. Non-parametric tests were used to analyze the correlations between two unordered categorical variables. The corrplot package was applied to analyze the correlation between mutated genes by spearman bivariate correlation analysis. Chi-square tests were used in the analysis of mutated genes and lymph node metastasis, as well as the co-mutation status of gene alterations. To ensure the consistency of experimental results, each experiment was performed in triplicate and unpaired two-tailed Student’s t test was used for statistical analysis of the experiments. Statistical significance was considered when *p* < 0.05.

## Results

### Pathogenically high-risk variants among 31 driver genes and non-driver genes were detected in both cancer tissues and benign thyroid nodules

**Fig. 1:**
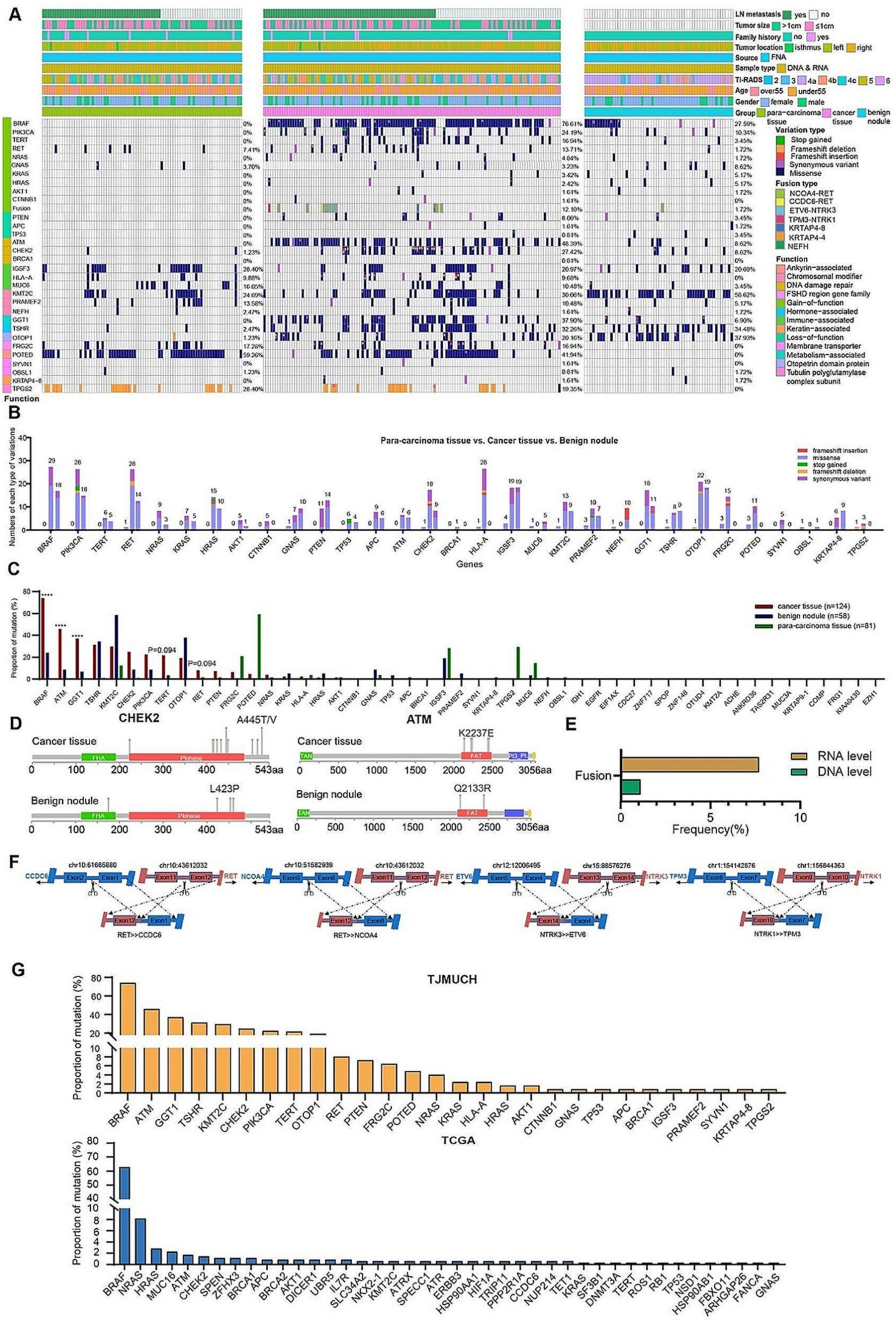
Profiles of genetic alterations significantly differ between thyroid carcinomas and benign nodules. **(A)** Overall analysis of gene alteration profiles, indicating that most mutated genes have significantly higher mutation ratios in cancer tissues than benign nodules. **(B)** The number of each particular type of variations in detected genes. **(C)** Mutation frequencies of all genes sequenced among cancer tissues, benign nodules and para-carcinoma tissues. **(D)** Mutation sites of genes in DNA damage repair signaling pathways. **(E)** Significantly higher frequency of gene fusions detected at the RNA level than the DNA level. **(F)** Fusion sites of the four fusion types: CCDC6-RET (2/124, 1.61%) is the fusion of CCDC6 exon 1 and RET exon 12, NCOA4-RET (6/124, 4.84%) is the fusion of NCOA4 exon 8 and RET exon 12, ETV6-NTRK3 (5/124, 4.03%) is the fusion of ETV6 exon 4 and NTRK3 exon 14, and TPM3-NTRK1 (1/124, 0.81%) is the fusion of TPM3 exon 7 and NTRK1 exon 10. **(G)** Mutation proportion of mutated genes in our cohort and TCGA database

In total, 592 mutations were detected, including missense mutations (426/592, 71.96%), frame-shift insertions (27/592, 4.56%), frame-shift deletions (17/592, 2.87%), nonsense mutations (6/592, 1.01%) and synonymous variant (116/592, 19.59%). The pathogenic loci were further screened with ClinVar, and the pathogenicity of mutation sites was predicted to belong to variations of uncertain significance, among which probably harmful (115/476, 24.16%) and possibly harmful (57/476, 11.97%) loci were denoted pathogenically high-risk variants. A total of 172 pathogenically high-risk variants were detected, including somatic mutations and germline mutations. These variants were distributed among 31 genes in two major categories: driver genes (13 genes) and non-driver genes (18 genes). Driver genes included gain-of-function oncogenes (*BRAF*, *RET*, *CTNNB1*, *NRAS*, *HRAS*, *KRAS*, *AKT1*, *PIK3CA*, *TERT* and *GNAS*) and loss-of-function oncogenes (*TP53*, *PTEN* and *APC*). Non-driver genes included DNA damage repair genes (*CHEK2*, *ATM* and *BRCA1*), immune-associated genes (*HLA-A*, *MUC6* and *IGSF3*), chromosomal modifier genes (*KMT2C*, *PRAMEF2* and *NEFH*), membrane transporter-associated genes (*OBSL1* and *SYVN1*), hormone-associated genes (*TSHR*), metabolism-associated genes (*GGT1*), keratin-associated genes (*KRTAP4-8*), ankyrin-associated genes (*POTED*), genes of the otopetrin domain protein family (*OTOP1*), an FSHD region gene family member (*FRG2C*) and a tubulin polyglutamylase complex subunit (*TPGS2*) (Fig. 1A, B).

To further explore the presence of germline variations, mutations were screened with the conditions that variant allele frequency was over 40% and mutations were present in both cancer tissues and para-carcinoma tissues. *RET* mutation (c. G2071A: p.G691S) was detected, which suggested the possibility of hereditary medullary thyroid carcinoma. Besides, germline mutations in *POTED*, *TPGS2*, *IGSF3*, *KMT2C*, *FGR2C*, *MUC6*, *PRAMEF2, HLA-A*, *GNAS, OBSL1, OTOP1, NEFH, TSHR* and *CHEK2* were also detected (Table S5). Sequencing data for cancer tissues and benign nodules were filtered with reference to para-carcinoma tissues. And a total of 156 somatic mutations were detected, among which the top ten most frequently mutated genes were *BRAF*, *ATM*, *GGT1*, *TSHR*, *KMT2C*, *CHEK2*, *PIK3CA*, *TERT, OTOP1* and *RET*.

Furthermore, we compared the mutation profiles between cancer tissues and benign nodules. *BRAF*^V600E^ occurred more frequently in cancer tissues than benign nodules (92/124, 74.19% vs. 10/58, 17.24%, *p* < 0.0001), whereas *BRAF* mutations at other loci (such as the Raf-like Ras-binding domain) were detected primarily in benign nodules (10/58, 17.24% vs. 1/124, 0.81%, *p* < 0.0001). Mutations in *TERT* gene were dispersed over the entire sequence, but occurred primarily in the telomerase ribosomal nuclear protein complex-RNA binding domain (25/124, 20.16%). The frequency of *TERT* gene mutations was significantly higher in cancer tissues than benign nodules (27/124, 21.77% vs. 2/58, 3.45%, *p* = 0.094), thus suggesting the value of this gene in distinguishing benign from malignant thyroid nodules. Moreover, *RET* mutations accounted for a significantly higher proportion of mutations in cancer tissues than benign nodules (10/124, 8.06% vs. 1/58, 1.72%, *p* = 0.094). Furthermore, the mutation frequencies of DNA damage repair genes *ATM* and metabolism-associated genes *GGT1* were significantly higher in cancer tissues than benign nodules (Fig. 1C). Furthermore, more mutation sites of genes in DNA damage repair signaling were observed in cancer tissues than benign nodules (Fig. 1D).

Gene fusion is another gene alteration type in thyroid carcinoma [28–34]. Gene fusions were detected at both the RNA and DNA levels. At the DNA level, 2 patients (2/124, 1.61%) had fusions in *KRTAP4-8*, *KRTAP4-4* and *NEFH*. *CCDC6-RET* (2/124, 1.61%), *NCOA4-RET* (6/124, 4.84%), *ETV6-NTRK3* (5/124, 4.03%) and *TPM3-NTRK1* (1/124, 0.81%) were detected at the RNA level, and 13 were patients with cancer tissues (Fig. 1E). And fusions were the most common fusion types reported in previous studies (Fig. 1F).

### Comparison of mutation profiles of PTC tissues between in-house and TCGA data

Mutated genes were compared between cancer tissues and the thyroid carcinoma cohort from The Cancer Genome Atlas (TCGA) database. The mutation frequencies in *BRAF, NRAS*, *CHEK2*, *ATM, NRAS and HRAS* were relatively high in both TCGA cohort and our cohort. In addition to the high frequency mutated genes in TCGA, the mutation frequencies of *PIK3CA* were high in cancer tissues from our cohort, thus suggesting possible activation of the PI3k/Akt/mTOR pathway. Moreover, the mutation frequencies of *GGT1, TSHR*, *KMT2C, TERT, OTOP1* and *RET* were also relatively high in our cohort (Fig. 1G).

### Concomitant pathogenically high-risk variants are detected more frequently in PTC tissues than benign nodules and correlate with lymph node metastasis

**Fig. 2:**
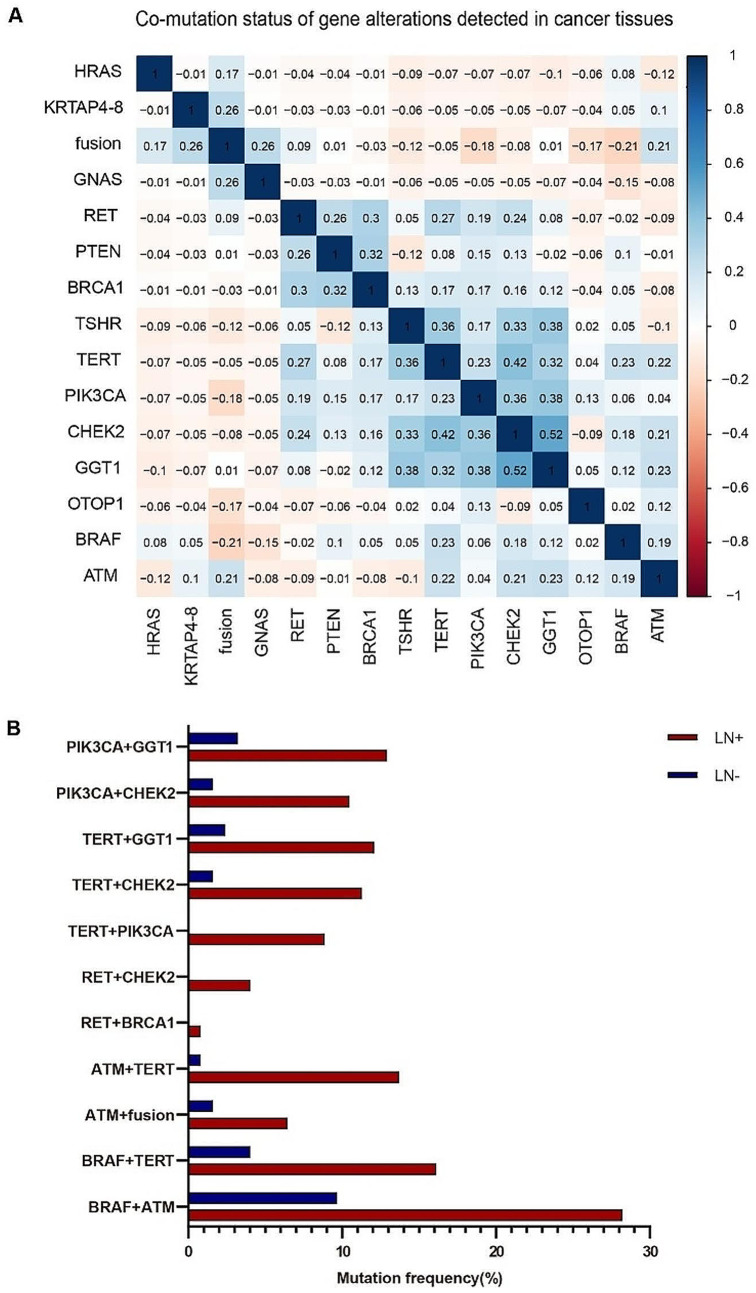
Concomitant pathogenically high-risk variants are detected more frequently in cancer tissues than benign nodules and are correlated with lymph node metastasis. **(A)** Analysis of correlations among mutated genes in cancer tissues. **(B)** Frequency of concomitant gene alterations in cancer tissues with or without lymph node metastasis

We analyzed the co-mutation status of gene mutations and fusions in PTC tissues. *BRAF*^V600E^ was concomitant with *ATM* (37.90%, *p* = 0.052), *TERT* (20.97%, *p* = 0.003) and *CHEK2* (23.39%, *p* = 0.004). *TERT* was co-mutated with *ATM* (14.52%, *p* = 0.025), *TSHR* (13.71%, *p* < 0.0001), *CHEK2* (12.9%, *p* < 0.0001), *RET* (4.03%, *p* = 0.024), *GGT1* (14.52%, *p* < 0.0001), *PIK3CA* (8.87%, *p* = 0.011) and *BRCA1* (0.81%, *p* = 0.057). *RET* was co-mutated with *PIK3CA* (4.03%, *p* = 0.031), *PTEN* (2.42%, *p* = 0.004), *AKT1* (0.81%, *p* = 0.028), *CHEK2* (4.03%, *p* = 0.057) and *BRCA1* (0.81%, *p* = 0.001). Furthermore, *ATM* (8.87%, *p* = 0.005), *GNAS* (0.81%, *p* = 0.003), *HRAS* (0.81%, *p* = 0.066) and *KRTAP4-8* (0.81%, *p* = 0.003) were frequently co-mutated with gene fusions (Fig. 2A).

Correlation analysis of gene alterations and lymph node metastasis (LN) showed that *RET*^MUT^ (*p* = 0.033), *ATM*^MUT^ (*p* = 0.031) and *TERT*^MUT^ (*p* = 0.005) were positively correlated with lymph node metastasis. Meanwhile, although the correlation between lymph node metastasis and *BRAF*^*V600E*^ (*p* = 0.136), as well as *PIK3CA*^*MUT*^ (*p* = 0.103), was not significant, they were also likely to be associated with lymph node metastasis (Table S6). We further analyzed the correlation between the genes above co-mutant with other genes and lymph node metastasis. *BRAF*^V600E^ and *ATM*^MUT^ or *TERT*^MUT^ were highly correlated with lymph node metastasis (*BRAF*^V600E^ and *ATM*^MUT^, *p* = 0.004; *BRAF*^V600E^ and *TERT*^MUT^, *p* = 0.013). *TERT*^MUT^ concomitant with *ATM*^MUT^, *CHEK2*^MUT^, *GGT1*^MUT^ and *PIK3CA*^MUT^ was highly associated with lymph node metastasis (*TERT*^MUT^ and *ATM*^MUT^, *p* = 0.001; *TERT*^MUT^ and *CHEK2*^MUT^, *p* = 0.011; *TERT*^MUT^ and *GGT1*^MUT^, *p* = 0.019; *TERT*^MUT^ and *PIK3CA*^MUT^, *p* = 0.003). *PIK3CA*^MUT^ concomitant with *GGT1*^MUT^ and *CHEK2*^MUT^ was highly associated with lymph node metastasis (*PIK3CA*^MUT^ and *GGT1*^MUT^, *p* = 0.03; *PIK3CA*^MUT^ and *CHEK2*^MUT^, *p* = 0.017). Concomitant *RET*^MUT^ and *CHEK2*^MUT^ were highly correlated with lymph node metastasis (*p* = 0.052). Besides, concomitant *ATM*^MUT^ and gene fusions were correlated with lymph node metastasis, with marginal significance (*p* = 0.143) (Fig. 2B). Therefore, co-mutations of driver genes had better performance than single mutations in predicting lymph node metastasis.

### *ATM* deficiency in PTC indicates high proliferation and aggressiveness of carcinoma cells

We classified 31 mutant genes in pathway enrichment analysis. A total of 11 pathways were enriched: thyroid hormone signaling, apoptosis, mTOR signaling, PI3K-Akt signaling, autophagy, VEGF signaling, MAPK signaling, p53 signaling, cAMP signaling, Ras signaling and homologous recombination (Figure S3A). In the analysis of the frequencies of mutated genes in each pathway, the mutation rates of genes in apoptosis (*p* < 0.0001), cAMP signaling (*p* < 0.0001), mTOR signaling (*p* < 0.0001), homologous recombination ( *p* < 0.0001), p53 signaling (*p* < 0.0001) and MAPK signaling (*p* < 0.0001) were significantly higher in cancer tissues than benign nodules (Figure S3B).

**Fig. 3:**
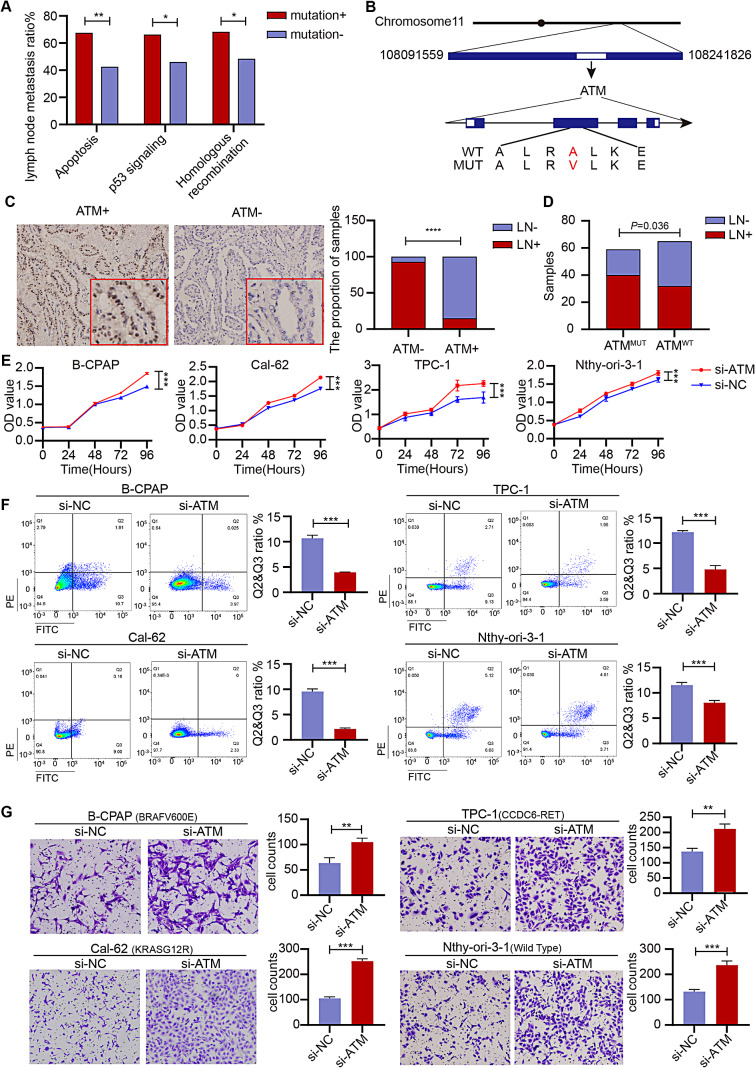
Deficiency in the homologous recombination repair gene ATM predicts elevated proliferation and aggressiveness in thyroid carcinoma. **(A)** Mutations in genes associated with apoptosis, the p53 signaling pathway and the homologous recombination pathway are more frequent in tumors with lymph node metastasis. **(B)** Mutation sites of ATM are focused in the FAT domain. **(C)** Low expression of ATM is relatively more frequent in samples with ATM^MUT^ and is significantly associated with lymph node metastasis. **(D)** ATM mutation was correlated with lymph node metastasis in cancer tissues. **(E)** Significantly greater relative viability of B-CPAP, TPC-1, Cal-62 and Nthy-ori-3-1 was shown after ATM knock-down than in NC-transfected cells. **(F)** Significantly decreased apoptosis was shown after decreasing ATM expression. **(G)** Significantly greater invasive capability of B-CPAP^si−ATM^, Cal-62^si − ATM^, TPC-1^si − ATM^ and Nthy-ori-3-1^si − ATM^ was shown than NC-transfected cells

Furthermore, we simultaneously conducted pathway enrichment analysis between tumors with and without lymph node metastasis. Genes associated with apoptosis (*p* = 0.002), p53 signaling (*p* = 0.022) and homologous recombination (*p* = 0.021) were mutated more frequently in tumors with than without lymph node metastasis (Fig. 3A). The homologous recombination repair gene *ATM* existed in all three pathways and correlated with lymph node metastasis. In the cohort, we identified a new *ATM* gene mutation absent from TCGA database. The mutation was located in the FAT domain, a unique motif at the extreme C terminus in PIK-related family members (Fig. 3B), and was predicted to be pathogenic. The formalin fixed paraffin embedded samples of 48 cancer tissues were collected and stained to detect *ATM* protein expression. Low expression of *ATM* was observed frequently in samples with *ATM*^MUT^ and was significantly associated with lymph node metastasis (Fig. 3C). And *ATM* mutation was highly correlated with lymph node metastasis in cancer tissues (Fig. 3D).

To explore the effects of *ATM* on the pro-tumoral effects of pathogenic mutations in driver genes, we established *ATM* knock-down thyroid cancer cell lines (B-CPAP, TPC-1, Cal-62 and Nthy-ori-3-1) (Figure S4). Compared with NC-transfected cells, B-CPAP, TPC-1, Cal-62 and Nthy-ori-3-1 cells had significantly increased viability after knock-down of *ATM* expression (Fig. 3E). After knock-down of *ATM* expression, the number of apoptotic cells significantly decreased (B-CPAP vs. B-CPAP^si−*ATM*^ = 10.69% ± 0.48% vs. 3.96% ± 0.06%, *p* < 0.0001; Cal-62 vs. Cal-62^si − *ATM*^ = 9.56% ± 0.42% vs. 2.18% ± 0.13%, *p* < 0.0001; TPC-1 vs. TPC-1^si − *ATM*^ = 12.15% ± 0.25% vs. 4.81% ± 0.62%, *p* = 0.0001; Nthy-ori-3-1 vs. Nthy-ori-3-1^si − *ATM*^ = 11.54% ± 0.43% vs. 8.06% ± 0.36%, *p* = 0.0009; Fig. 3F). The invasiveness of cells was also explored. The invasive ability of B-CPAP^si−*ATM*^, Cal-62^si − *ATM*^, TPC-1^si − *ATM*^ and Nthy-ori-3-1^si − *ATM*^ cells was significantly greater than that of NC-transfected cells (B-CPAP vs. B-CPAP^si−NC^ vs. B-CPAP^si−*ATM*^ = 56.33 ± 4.5 vs. 63.33 ± 8.73 vs. 105 ± 6.53, *p* = 0.0057; Cal-62 vs. Cal-62^si − NC^ vs. Cal-62^si − *ATM*^ = 104.67 ± 4.99 vs. 105.33 ± 4.64 vs. 212.33 ± 7.85, *p* < 0.0001; TPC-1 vs. TPC-1^si − NC^ vs. TPC-1^si − *ATM*^ = 163 ± 8.6 vs. 137.33 ± 8.65 vs. 212 ± 13.14, *p* = 0.0026; Nthy-ori-3-1 vs. Nthy-ori-3-1^si − NC^ vs. Nthy-ori-3-1^si − *ATM*^ = 137 ± 12.33 vs. 132 ± 6.68 vs. 236.67 ± 13.27, *p* = 0.0026; Fig. 3G). Thus, *ATM* deficient thyroid cancer cells bearing *BRAF*^V600E^, *KRAS*^G12R^ or *CCDC6-RET* displayed higher proliferative ability, lower apoptotic rate and greater aggressiveness than NC-transfected cells *in vitro.*

### Combining gene alterations and clinical features with ultrasonographic radiomics significantly improves the predictive efficacy for lymph node metastasis in PTC

**Fig. 4:**
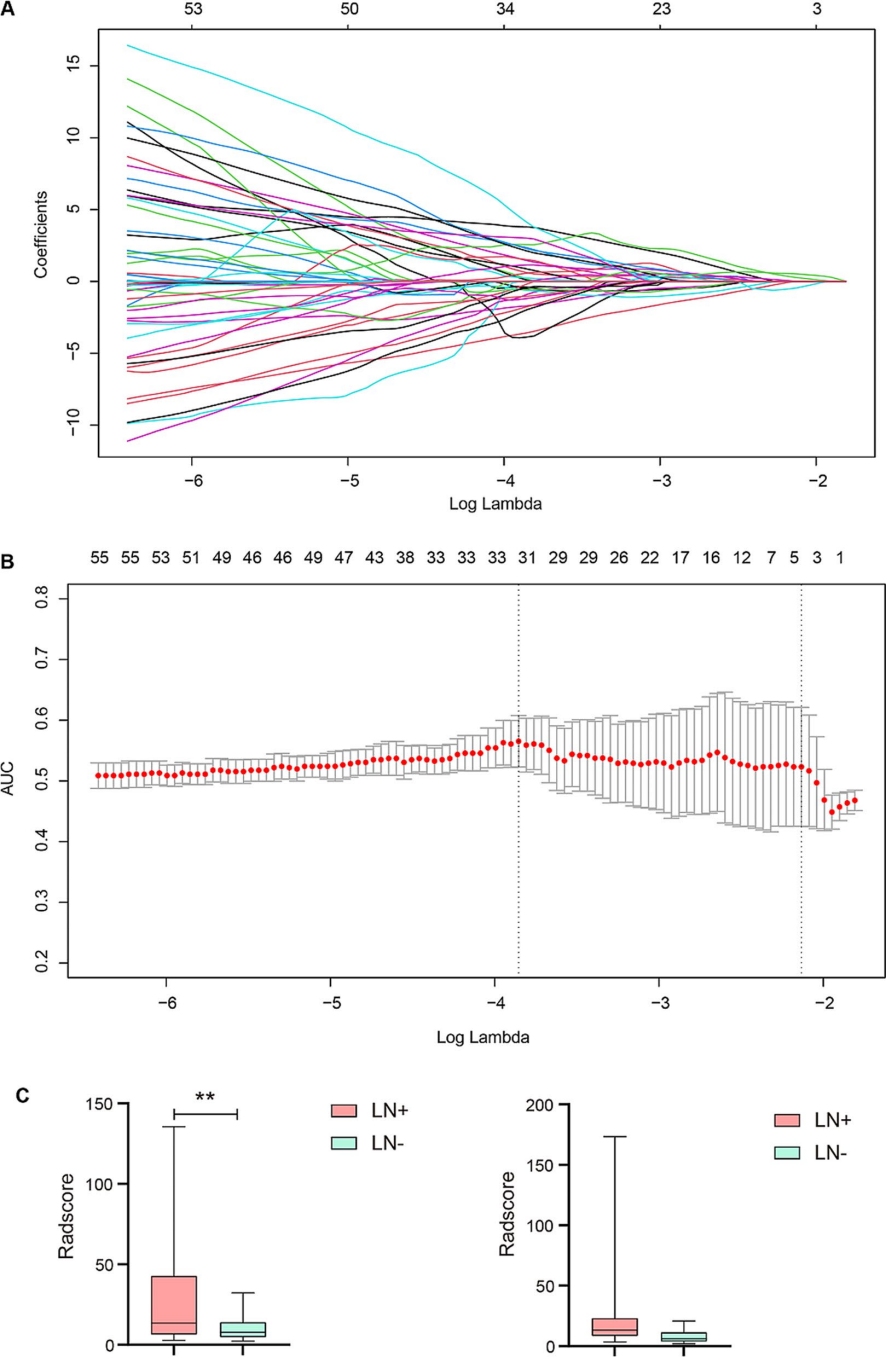
Feature selection using the least absolute shrinkage and selection operator (LASSO) algorithm. **(A)** A coefficient path plot was generated showing how the coefficients of each variable changed at different regularization levels. **(B)** AUC (red dots) with standard errors (error bar) can be used to determine the optimum penalty lambda (λ). **(C)** Boxplot of Radscores between cancer tissues with or without lymph node metastasis from the training set (left) and the test set (right)

To explore the effect of radiomics on predicting lymph node metastasis in our cohort, we extracted 854 radiomic features from 108 PTC samples. The Glmnet package was used to filter the smallest regularization parameter lambda. The model was fitted by minimizing a combination of the loss function and the regularization term (Fig. 4A). Area under curve (AUC) with standard errors can be used to determine the optimum penalty lambda (λ) by cross-validation (Fig. 4B). Three features (wavelet-LHL_glrlm_Short Run Emphasis, wavelet-HLH_glszm_Size Zone Non-Uniformity and wavelet-HLH_glszm_Small Area Low Gray Level Emphasis) associated with lymph node metastasis were identified, and a radiomics signature model for predicting lymph node metastasis of PTC was constructed integrating these three features. Furthermore, a radiomics score (Radscore) was calculated for each sample based on the features selected. Cancer tissues with lymph node metastasis had significantly higher Radscores than those without lymph node metastasis in the training set, and cancer tissues with lymph node metastasis also tended to have higher Radscores in the test set (Fig. 4C, Table S7).

**Fig. 5:**
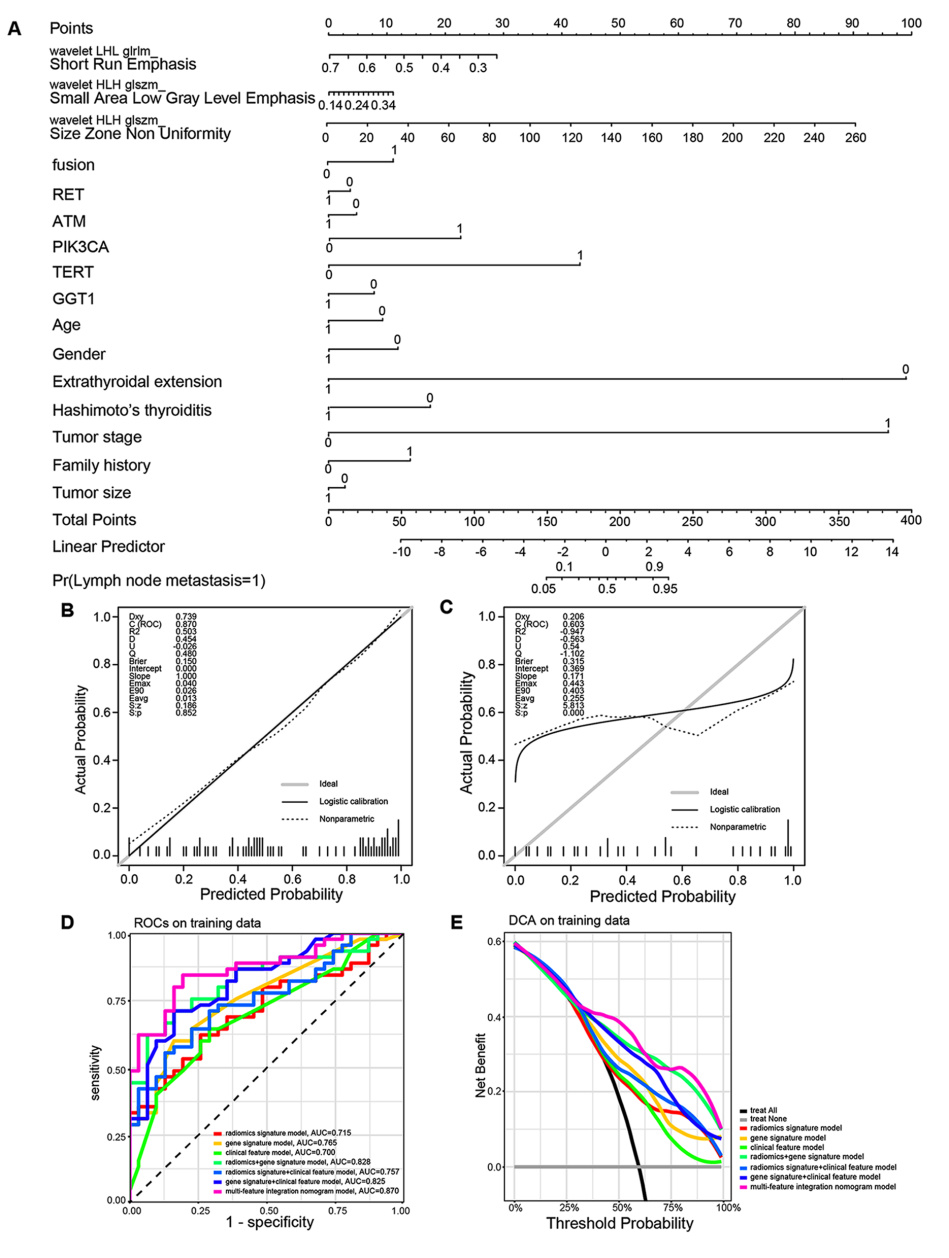
Construction of a multi-feature integration nomogram model and comparison of the seven models. **(A)** The multi-feature integration nomogram model, integrating radiomic features, mutated genes and clinical features, developed in the training set. **(B)** Calibration curves for the multi-feature integration nomogram model in the training set. **(C)** Calibration curves for the multi-feature integration nomogram model in the test set. **(D)** ROC curves of the seven models in the training set. **(E)** DCAs for the seven models in the training set

Next, we explored the efficacy of gene alterations, clinical features and radiomics in the prediction of lymph node metastasis. Mutated genes associated with lymph node metastasis in the univariate analysis, including *RET, ATM, PIK3CA, TERT, GGT1* and fusions, were incorporated to construct a gene signature model. And a clinical feature model based on clinical characteristics (including age, gender, extrathyroidal extension, Hashimoto’s thyroiditis, tumor stage, family history and tumor size) was also constructed. The multi-feature integration nomogram model was then optimized integrating gene alterations, clinical features and radiomic features (Fig. 5A). The calibration plots of multi-feature integration nomogram model showed good calibration in the training set (Fig. 5B, C). We compared the lymph node metastasis prediction efficacy of the gene signature model, clinical feature model, radiomics signature model, their pairwise combination and the multi-feature integration nomogram model. The AUC of the multi-feature integration nomogram model (0.8695, 95% CI:0.7909–0.9482) was higher than that of the gene signature model (0.7649, 95% CI: 0.6614–0.8683), clinical feature model (0.7, 95% CI: 0.5826–0.8174), and radiomics signature model (0.7147, 95% CI: 0.6003–0.8291), as well as their pairwise combinations (Fig. 5D, Figure S5A, Table 1). These findings indicated that combining gene alterations and clinical features with radiomic signatures increased the efficacy of the model for predicting lymph node metastasis of thyroid carcinoma. The Decision Curve Analysis (DCAs) of the training data based on the seven models are presented in Fig. 5E (DCAs of the test data are presented in Figure S5B). For the prediction of lymph node metastasis of PTC, the multi-feature integration nomogram model had the highest overall net benefit.

**Table 1:** The AUC of models predicting lymph node metastasis.

Models of lymph node metastasis prediction	Training set (AUC; 95% CI)	Test set (AUC; 95% CI)
gene signature model	0.7649; 0.6614-0.8683	0.5891; 0.386-0.7921
clinical feature model	0.7; 0.5826-0.8174	0.6619; 0.447-0.8769
radiomics signature model	0.7147; 0.6003-0.8291	0.6721; 0.4787-0.8654
gene signature model + clinical feature model	0.8247; 0.7323-0.9172	0.6235; 0.417-0.83
gene signature model + radiomics signature model	0.828; 0.7362-0.9197	0.6356; 0.4361-0.8351
clinical feature model + radiomics signature model	0.757; 0.6502-0.8638	0.6559; 0.45-0.8617
multi-feature integration nomogram model	0.8695; 0.7909-0.9482	0.6032; 0.4005-0.8059

## Discussion

Driver gene variations have important roles in thyroid carcinoma initiation and development, and gene mutations and fusions are the most common genetic variation types in PTC. Genetic alterations are likely to be missed by conventional genetic testing methods, such as PCR assays, when they are present at unknown loci at low frequency or in limited quantities of FNA samples [35, 36]. Therefore, NGS-based DNA sequencing has been widely applied for the clinical genetic testing of thyroid carcinoma, because of its high throughput and speed. However, as a growing number of fusion types in thyroid carcinoma have been demonstrated to promote carcinogenesis and to be actionable targets of multiple tyrosine kinase inhibitors [37], detection of gene fusions is becoming an indispensable genetic examination. Given that gene fusions are frequently undetectable with DNA sequencing, studies have focused on investigating RNA sequencing in cancer molecular diagnosis. In this study, gene fusions were detected in 7.69% (14/182) of RNA samples but only 1.10% (2/182) of DNA samples, thus suggesting that targeted sequencing of DNA/RNA is more sensitive and accurate for gene fusion detection than DNA sequencing alone.

All samples in the study were collected through FNA. Compared with surgical tissue specimens, the relatively small amount of tissue in FNA samples might have affected the quantity of nucleic acids. Moreover, the possibility of puncturing non-tumor areas, insufficient pre-sampling preparations and delayed post-sampling handling could influence the accuracy of sequencing. However, because of the low amount of nucleic acid required for NGS, a minimum of 10 ng nucleic acid met the library construction requirements. To ensure the tissue quantity and quality of FNA samples, the senior radiologist (Dr. X. Wei, with 15 years of experience in thyroid carcinoma ultrasound diagnosis) performed sample puncture under ultrasound monitoring, avoiding necrotic tissue and vascular-rich areas to minimize bleeding. In addition, we prepared nucleic acid-free containers and liquid nitrogen tanks before puncture, and the samples were immediately processed after puncture to avoid nucleic acid degradation and to ensure the integrity of nucleic acids in FNA tissues. The tumor proportion of FNA samples was evaluated by two experienced pathologists and was at least 20%, to fully reflect the patients’ tumor condition. Hence, the quality of sequencing and the accuracy of data interpretation were assured.

Integrating FNA outcomes with other molecular markers, such as *BRAF* mutations, has been reported to be essential for precise categorization of PTC [38]. Basing categorization solely on FNA results for benign nodules might introduce bias; consequently, the ten benign nodules identified to bear *BRAF*^V600E^ mutation might have been cancer tissues. Therefore, we reanalyzed 182 samples by using a new grouping strategy (134 cancer tissues vs. 48 benign nodules), and found no significant difference in genetic mutation profiles between the grouping strategies (Figure S6).

Because thyroid carcinoma involves multiple genetic alterations, single gene alterations cannot accurately predict the prognosis of thyroid carcinoma. We demonstrated that driver genes (*BRAF*, *RET* and *TERT*, etc.) are co-mutated with other genes, such as DNA damage repair genes and metabolism related genes, thus forming a co-mutation pattern observed more frequently in cancer tissues than benign nodules. Moreover, concomitant mutation patterns predicted lymph node metastasis better than single gene alterations, thus suggesting that, for patients with PTC with detectable driver gene mutations, the alterations in non-driver genes, such as DNA damage repair genes, warrant close attention, to enable more accurate and efficient prediction of lymph node metastasis.

*ATM* was the most common homologous recombination repair gene co-mutated with driver genes. Single nucleotide polymorphisms in *ATM* have been detected in patients exposed to radiation, and play an important role as a modifier of PTC risk associated with *BRCA1* and *CHEK2* [39]. *ATM* mutations in this cohort were focused in the FAT domain of *ATM*, as reported in other studies [40–43]. Impaired function of *ATM* can lead to dysfunctional DNA damage repair, thereby increasing chromosome instability and gene alterations [44–46]. Moreover, loss-of-function mutation of *ATM* inhibits the downstream p53 apoptotic pathway, thus promoting cell survival [47–57]. Consistently, cell assays in vitro showed that cancer cells with driver genetic alterations accompanied by *ATM* deficiency had high capability in proliferation and invasion, thus suggesting that *ATM* may assist driver oncogenes in enhancing tumor-promoting effects.

Ultrasound is an important pretreatment risk assessment modality for patients with PTC. Here, ultrasound-based radiomic features, accompanied by gene alterations and clinical features, were applied to construct a model to predict lymph node metastasis of PTC. We used feature selection techniques to minimize the number of genes integrated; and we additionally incorporated clinical features and mutated genes related to lymph node metastasis, including *RET, ATM, PIK3CA, TERT, GGT1* and fusions, to construct the prediction model for lymph node metastasis of PTC [58]. We compared the predictive efficacy between the model including all mutated genes and the model including lymph node metastasis related genes. Although the AUC of the model including all mutated genes was higher than that of the model including lymph node metastasis related genes (0.8989, 95% CI: 0.8333–0.9646 vs. 0.828, 95% CI: 0.7362–0.9197), both models displayed higher predictive efficacy than the radiomics signature model (Figure S7). Furthermore, clinical features, including age, gender, extrathyroidal extension, Hashimoto’s thyroiditis, tumor stage, family history and tumor size, were also incorporated to construct the multi-feature integration nomogram model. It should be noted that potential interactions or collinearity might exist among the predictors included in the model, which affect β coefficient of the model and lead to unreliable effect estimates. Thus, in the process of model construction, it is important to analyze the collinearity of predictors included in the model.

The model prediction results showed that gene alterations, clinical features and radiomic signatures alone had limited ability to predict lymph node metastasis, whereas combining gene alterations and clinical features with radiomic signatures was more effective in predicting lymph node metastasis of PTC. Further evaluation indicated that the performance of the prediction model combining clinical and genetic features was as efficient as that of the model combining genetic and radiomic features. However, the predictive efficacy of the combination of clinical and radiomic features toward lymph node metastasis was relatively lower, thus potentially leading to false negative diagnosis. Therefore, in pretreatment risk assessment for patients with PTC, gene alteration detection could be combined with clinical features and ultrasound-based radiomics to predict lymph node metastasis more accurately and efficiently, and assist in determining treatment options.

In the process of determining lambda values, we observed that the forecast confidence interval suddenly, possibly because of the small size of the dataset and the heterogeneity of the clinical samples. In the test set, we observed that the AUC value of the multi-feature integration nomogram model was lower than that of the individual clinical or radiomics feature models, possibly because the limited samples in the test set affected the robustness of the model. Similarly, the presented calibration curves indicated strong performance in the training set, whereas a significant divergence was observed in the test set, thus suggesting overfitting to the training data. To address overfitting, approaches such as cross-validation and regularization should be adopted in the model. Moreover, a machine learning algorithm for a prognostic prediction model based on clinical features, radiomic features and genetic features should be established to improve predictive accuracy. More PTC samples should also be studied to assess the model’s performance, and a multi-center clinical trial should be used as an independent validation set to evaluate generalizability. Thus, a more accurate prediction model could be developed for clinical application.

**Fig. 6:**
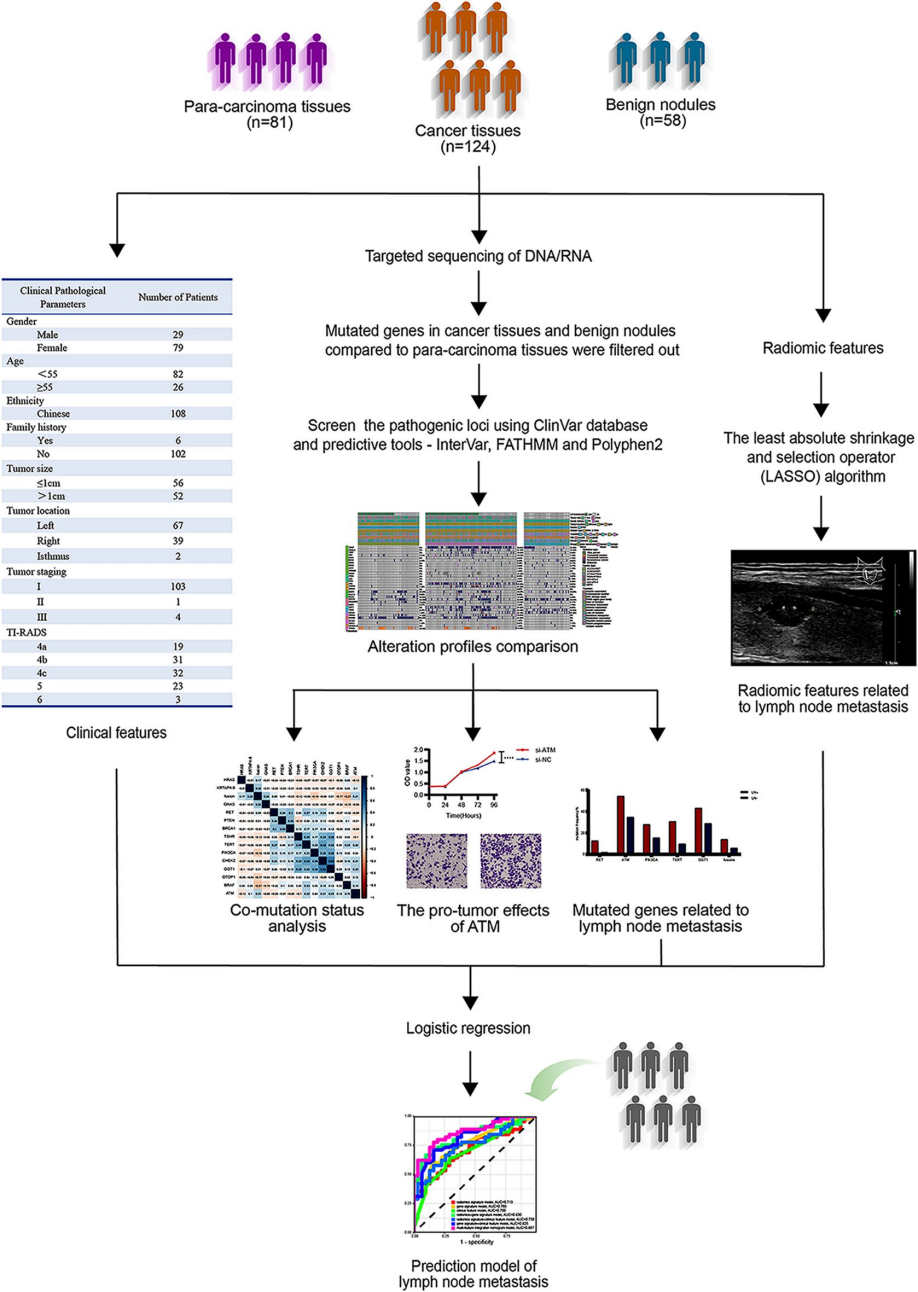
Graphical overview. NGS-based targeted sequencing of DNA/RNA for 124 cases of cancer tissues, 58 cases of benign nodules and 81 cases of para-carcinoma tissues were conducted. Mutated genes in cancer tissues and benign nodules compared to para-carcinoma tissues were filtered out. The pathogenic loci were screen using ClinVar database and predictive tools - InterVar, FATHMM and Polyphen2. Mutation profiles were compared between cancer tissues and benign nodules, as well as the thyroid carcinoma cohort from The Cancer Genome Atlas (TCGA) database. Further, co-mutation status analysis of the gene alterations was conducted in cancer tissues and a multiple-gene co-mutation pattern associated with lymph node metastasis was described. Besides, a multi-feature integration nomogram model incorporating gene alterations, clinical features and radiomics to predict lymph node metastasis by LASSO-logistic regression method

In conclusion, we used targeted sequencing of DNA/RNA to identify new gene markers for predicting the prognostic risk of PTC. A multiple-gene co-mutation pattern associated with lymph node metastasis was described. We also established a multi-feature integration nomogram model incorporating gene alterations, clinical features and radiomics to predict lymph node metastasis more precisely. In brief, we strongly recommend multiple-gene alteration analysis for patients with PTC in clinical practice, to comprehensively assess the potential risk of lymph node metastasis and more precisely determine options for clinical treatment (Fig. 6).

### Electronic supplementary material

Below is the link to the electronic supplementary material.

Supplementary Material 1

Supplementary Material 2

Supplementary Material 3

## Data Availability

The data generated in this study are available upon request from the corresponding author.
