# Supplementary material for: Targeted sequencing of DNA/RNA combined with radiomics predicts lymph node metastasis of papillary thyroid carcinoma
Source: Cancer Imaging. 2024 Jun 17;24:75. doi: 10.1186/s40644-024-00719-2 (PMC11181663; doi:10.1186/s40644-024-00719-2)
Supplement: Supplementary file 1 — Supplementary Material 1 [file 40644_2024_719_MOESM1_ESM.doc]

**Figure legends**

**Figure 1** Profiles of genetic alterations significantly differ between thyroid carcinomas and benign nodules. **A.** Overall analysis of gene alteration profiles, indicating that most mutated genes have significantly higher mutation ratios in cancer tissues than benign nodules. **B.** The number of each particular type of variations in detected genes. **C.** Mutation frequencies of all genes sequenced among cancer tissues, benign nodules and para-carcinoma tissues. **D.** Mutation sites of genes in DNA damage repair signaling pathways. **E.** Significantly higher frequency of gene fusions detected at the RNA level than the DNA level. **F.** Fusion sites of the four fusion types: CCDC6-RET (2/124, 1.61%) is the fusion of CCDC6 exon 1 and RET exon 12, NCOA4-RET (6/124, 4.84%) is the fusion of NCOA4 exon 8 and RET exon 12, ETV6-NTRK3 (5/124, 4.03%) is the fusion of ETV6 exon 4 and NTRK3 exon 14, and TPM3-NTRK1 (1/124, 0.81%) is the fusion of TPM3 exon 7 and NTRK1 exon 10. **G.** Mutation proportion of mutated genes in our cohort and TCGA database.

**Figure 2** Concomitant pathogenically high-risk variants are detected more frequently in cancer tissues than benign nodules and are correlated with lymph node metastasis. **A.** Analysis of correlations among mutated genes in cancer tissues. **B.** Frequency of concomitant gene alterations in cancer tissues with or without lymph node metastasis.

**Figure 3** Deficiency in the homologous recombination repair gene ATM predicts elevated proliferation and aggressiveness in thyroid carcinoma. **A.** Mutations in genes associated with apoptosis, the p53 signaling pathway and the homologous recombination pathway are more frequent in tumors with lymph node metastasis. **B.** Mutation sites of ATM are focused in the FAT domain. **C.** Low expression of ATM is relatively more frequent in samples with ATM^MUT^ and is significantly associated with lymph node metastasis. **D.** ATM expression negatively correlates with lymph node metastasis in cancer tissues. **E.** Significantly greater relative viability of B-CPAP, TPC-1, Cal-62 and Nthy-ori-3-1 was shown after ATM knock-down than in NC-transfected cells. **F.** Significantly decreased apoptosis was shown after decreasing ATM expression. **G.** Significantly greater invasive capability of B-CPAP^si-ATM^, Cal-62^si-ATM^, TPC-1^si-ATM^ and Nthy-ori-3-1^si-ATM^ was shown than NC-transfected cells.

**Figure 4** Feature selection using the least absolute shrinkage and selection operator (LASSO) algorithm. **A.** A coefficient path plot was generated showing how the coefficients of each variable changed at different regularization levels. **B.** AUC (red dots) with standard errors (error bar) can be used to determine the optimum penalty lambda (λ). **C.** Boxplot of Radscores between cancer tissues with or without lymph node metastasis from the training set (left) and the test set (right).

**Figure 5** Construction of a multi-feature integration nomogram model and comparison of the seven models. **A.** The multi-feature integration nomogram model, integrating radiomic features, mutated genes and clinical features, developed in the training set. **B.** Calibration curves for the multi-feature integration nomogram model in the training set. **C.** Calibration curves for the multi-feature integration nomogram model in the test set. **D.** ROC curves of the seven models in the training set. **E.** DCAs for the seven models in the training set.

**Figure 6** Graphical overview. NGS-based targeted sequencing of DNA/RNA for 124 cases of cancer tissues, 58 cases of benign nodules and 81 cases of para-carcinoma tissues were conducted. Mutated genes in cancer tissues and benign nodules compared to para-carcinoma tissues were filtered out. The pathogenic loci were screen using ClinVar database and predictive tools - InterVar, FATHMM and Polyphen2. Mutation profiles were compared between cancer tissues and benign nodules, as well as the thyroid carcinoma cohort from The Cancer Genome Atlas (TCGA) database. Further, co-mutation status analysis of the gene alterations was conducted in cancer tissues and a multiple-gene co-mutation pattern associated with lymph node metastasis was described. Besides, a multi-feature integration nomogram model incorporating gene alterations, clinical features and radiomics to predict lymph node metastasis by LASSO-logistic regression method.

**Figure S1** Representative ultrasound images of PTC primary tumors with different TI-RADS classification.

**Figure S2** Development of the multi-feature integration nomogram model for lymph node metastasis prediction.

**Figure S3** **A.** Pathway enrichment of mutated genes. **B.** Mutation frequencies of mutated gene related pathways in cancer tissues and benign nodules.

**Figure S4** **A.** Verification of interference effects of ATM at the RNA level. **B.** Verification of interference effects of ATM at the protein level.

**Figure S5 A.** ROC curves of the seven models in the test set. **B.** DCAs for the seven models in the test set.

**Figure S6 A.** Genetic mutation profiles of cancer tissues (134 cases) and benign nodules (48 cases). **B.** Differential mutated genes in cancer tissues (134 cases) and benign nodules (48 cases).

**Figure S7 A.** Calibration curves for the radiomics signature plus gene signature model including all mutated genes in the training set and the test set. **B.** ROC curves of the radiomics signature model and radiomics signature plus gene signature model including all mutated genes in the training set. **C.** Decision curve analysis for two models in the training set.
