# Supplementary material for: Targeted sequencing of DNA/RNA combined with radiomics predicts lymph node metastasis of papillary thyroid carcinoma
Source: Cancer Imaging. 2024 Jun 17;24:75. doi: 10.1186/s40644-024-00719-2 (PMC11181663; doi:10.1186/s40644-024-00719-2)
Supplement: Supplementary file 2 — Supplementary Material 2 [file 40644_2024_719_MOESM2_ESM.doc]

| **Table S6** Correlation analysis of gene alterations and lymph node metastasis   | Mutation | Lymph node metastasis | | P value | Mutation | Lymph node metastasis | | P value | | --- | --- | --- | --- | --- | --- | --- | --- | | Yes | No | Yes | No | | KMT2C |  |  |  | OTOP1 |  |  |  | | Yes | 17 | 20 | 0.075 | Yes | 12 | 12 | 0.373 | | No | 55 | 32 |  | No | 60 | 40 |  | | HLA-A |  |  |  | AKT1 |  |  |  | | Yes | 2 | 1 | 0.76 | Yes | 2 | 0 | 0.226 | | No | 70 | 51 |  | No | 70 | 52 |  | | BRAF |  |  |  | GGT1 |  |  |  | | Yes | 57 | 35 | 0.136 | Yes | 31 | 15 | 0.106 | | No | 15 | 17 |  | No | 41 | 37 |  | | RET |  |  |  | PRAMEF2 |  |  |  | | Yes | 9 | 1 | 0.033 | Yes | 0 | 1 | 0.237 | | No | 63 | 51 |  | No | 72 | 51 |  | | ATM |  |  |  | TPGS2 |  |  |  | | Yes | 39 | 18 | 0.031 | Yes | 0 | 1 | 0.237 | | No | 33 | 34 |  | No | 72 | 51 |  | | TSHR |  |  |  | IGSF3 |  |  |  | | Yes | 23 | 16 | 0.889 | Yes | 1 | 0 | 0.394 | | No | 49 | 36 |  | No | 71 | 52 |  | | APC |  |  |  | BRCA1 |  |  |  | | Yes | 0 | 1 | 0.237 | Yes | 1 | 0 | 0.394 | | No | 72 | 51 |  | No | 71 | 52 |  | | PIK3CA |  |  |  | SYVN1 |  |  |  | | Yes | 20 | 8 | 0.103 | Yes | 1 | 0 | 0.394 | | No | 52 | 44 |  | No | 71 | 52 |  | | GNAS |  |  |  | TERT |  |  |  | | Yes | 0 | 1 | 0.237 | Yes | 22 | 5 | 0.005 | | No | 72 | 51 |  | No | 50 | 47 |  | | CHEK2 |  |  |  | POTED |  |  |  | | Yes | 20 | 11 | 0.401 | Yes | 2 | 4 | 0.208 | | No | 52 | 41 |  | No | 70 | 48 |  | | HRAS |  |  |  | FRG2C |  |  |  | | Yes | 1 | 1 | 0.816 | Yes | 4 | 4 | 0.633 | | No | 71 | 51 |  | No | 68 | 48 |  | | KRAS |  |  |  | TP53 |  |  |  | | Yes | 1 | 2 | 0.38 | Yes | 0 | 1 | 0.237 | | No | 71 | 50 |  | No | 72 | 51 |  | | NARS |  |  |  | PTEN |  |  |  | | Yes | 3 | 2 | 0.929 | Yes | 6 | 3 | 0.587 | | No | 69 | 50 |  | No | 66 | 49 |  | | KRTAP4-8 |  |  |  | fusion |  |  |  | | Yes | 1 | 0 | 0.394 | Yes | 10 | 3 | 0.145 | | No | 71 | 52 |  | No | 62 | 49 |  |   **Table S7** Radscore and lymph node metastasis of each sample in the training set and testing set | | | | | |
| --- | --- | --- | --- | --- | --- | --- | --- | --- | --- | --- | --- | --- | --- | --- | --- | --- | --- | --- | --- | --- | --- | --- | --- | --- | --- | --- | --- | --- | --- | --- | --- | --- | --- | --- | --- | --- | --- | --- | --- | --- | --- | --- | --- | --- | --- | --- | --- | --- | --- | --- | --- | --- | --- | --- | --- | --- | --- | --- | --- | --- | --- | --- | --- | --- | --- | --- | --- | --- | --- | --- | --- | --- | --- | --- | --- | --- | --- | --- | --- | --- | --- | --- | --- | --- | --- | --- | --- | --- | --- | --- | --- | --- | --- | --- | --- | --- | --- | --- | --- | --- | --- | --- | --- | --- | --- | --- | --- | --- | --- | --- | --- | --- | --- | --- | --- | --- | --- | --- | --- | --- | --- | --- | --- | --- | --- | --- | --- | --- | --- | --- | --- | --- | --- | --- | --- | --- | --- | --- | --- | --- | --- | --- | --- | --- | --- | --- | --- | --- | --- | --- | --- | --- | --- | --- | --- | --- | --- | --- | --- | --- | --- | --- | --- | --- | --- | --- | --- | --- | --- | --- | --- | --- | --- | --- | --- | --- | --- | --- | --- | --- | --- | --- | --- | --- | --- | --- | --- | --- | --- | --- | --- | --- | --- | --- | --- | --- | --- | --- | --- | --- | --- | --- | --- | --- | --- | --- | --- | --- | --- | --- | --- | --- | --- | --- | --- | --- | --- | --- | --- | --- | --- | --- | --- | --- | --- | --- | --- | --- | --- | --- | --- | --- | --- | --- | --- | --- | --- | --- | --- | --- | --- | --- | --- | --- | --- | --- | --- | --- | --- | --- | --- | --- | --- | --- | --- | --- | --- | --- | --- | --- | --- | --- | --- | --- | --- | --- | --- | --- | --- | --- | --- | --- | --- | --- | --- | --- | --- | --- | --- | --- | --- | --- | --- | --- | --- | --- | --- | --- | --- | --- | --- | --- | --- | --- | --- | --- | --- | --- | --- | --- | --- | --- | --- | --- | --- | --- | --- | --- | --- | --- | --- | --- | --- | --- | --- | --- | --- | --- | --- | --- | --- | --- | --- | --- | --- | --- | --- | --- | --- | --- | --- | --- | --- | --- | --- | --- | --- | --- | --- | --- | --- | --- | --- | --- | --- | --- | --- | --- | --- | --- | --- | --- | --- |
| Training set | | | | | |
| Samples | Radscore | Lymph node metastasis | Samples | Radscore | Lymph node metastasis |
| Patient1 | 6.973201126 | 0 | Patient39 | 6.403273179 | 1 |
| Patient2 | 6.782181273 | 0 | Patient40 | 12.06520683 | 0 |
| Patient3 | 13.35667131 | 1 | Patient41 | 4.070497592 | 0 |
| Patient4 | 53.81465066 | 1 | Patient42 | 14.4295359 | 0 |
| Patient5 | 9.979265507 | 0 | Patient43 | 13.50428603 | 1 |
| Patient6 | 24.04723296 | 1 | Patient44 | 26.37415738 | 1 |
| Patient7 | 10.82203758 | 0 | Patient45 | 5.412648093 | 1 |
| Patient8 | 96.32856544 | 1 | Patient46 | 6.863786084 | 1 |
| Patient9 | 6.730500321 | 1 | Patient47 | 7.883175216 | 1 |
| Patient10 | 7.389019034 | 0 | Patient48 | 44.70917041 | 1 |
| Patient11 | 3.340543262 | 1 | Patient49 | 9.177703351 | 0 |
| Patient12 | 3.206227701 | 1 | Patient50 | 21.13480653 | 1 |
| Patient13 | 19.95890896 | 1 | Patient51 | 14.42925475 | 0 |
| Patient14 | 2.87769119 | 1 | Patient52 | 5.725886473 | 0 |
| Patient15 | 20.82082178 | 0 | Patient53 | 54.69912851 | 1 |
| Patient16 | 2.258354363 | 0 | Patient54 | 4.333556209 | 1 |
| Patient17 | 9.446552303 | 1 | Patient55 | 5.478977489 | 1 |
| Patient18 | 17.87661901 | 1 | Patient56 | 2.910621255 | 1 |
| Patient19 | 5.819682911 | 0 | Patient57 | 2.779803503 | 1 |
| Patient20 | 30.04968214 | 1 | Patient58 | 10.17823396 | 0 |
| Patient21 | 32.31499203 | 0 | Patient59 | 16.5730261 | 1 |
| Patient22 | 8.853880732 | 1 | Patient60 | 8.946720589 | 0 |
| Patient23 | 53.25624474 | 1 | Patient61 | 14.30047449 | 1 |
| Patient24 | 7.06440226 | 0 | Patient62 | 3.611220229 | 0 |
| Patient25 | 80.23736076 | 1 | Patient63 | 16.94704995 | 0 |
| Patient26 | 4.05623648 | 0 | Patient64 | 5.074876543 | 0 |
| Patient27 | 31.5278613 | 0 | Patient65 | 9.951395077 | 0 |
| Patient28 | 14.02937952 | 0 | Patient66 | 7.838409372 | 0 |
| Patient29 | 13.05902038 | 1 | Patient67 | 9.116523539 | 1 |
| Patient30 | 76.45912684 | 1 | Patient68 | 83.41280339 | 1 |
| Patient31 | 55.88120782 | 1 | Patient69 | 3.864753505 | 1 |
| Patient32 | 22.04182924 | 1 | Patient70 | 40.79854991 | 1 |
| Patient33 | 7.678045092 | 1 | Patient71 | 2.862893685 | 0 |
| Patient34 | 8.463751602 | 1 | Patient72 | 4.783250673 | 0 |
| Patient35 | 6.570049398 | 1 | Patient73 | 3.461943583 | 0 |
| Patient36 | 16.26324797 | 0 | Patient74 | 135.4056733 | 1 |
| Patient37 | 2.654896815 | 0 | Patient75 | 77.79616336 | 1 |
| Patient38 | 5.93151023 | 1 | Patient76 | 16.02620073 | 1 |

| Test set | | | | | |
| --- | --- | --- | --- | --- | --- |
| Samples | Radscore | Lymph node metastasis | Samples | Radscore | Lymph node metastasis |
| Patient1 | 13.19351158 | 1 | Patient17 | 23.43704515 | 1 |
| Patient2 | 4.461820831 | 1 | Patient18 | 173.3662581 | 1 |
| Patient3 | 3.94901142 | 0 | Patient19 | 22.27211058 | 1 |
| Patient4 | 3.420085968 | 1 | Patient20 | 14.96128342 | 0 |
| Patient5 | 7.892706552 | 0 | Patient21 | 10.73223233 | 1 |
| Patient6 | 14.94307059 | 1 | Patient22 | 7.180193977 | 0 |
| Patient7 | 5.300769039 | 0 | Patient23 | 20.68580163 | 0 |
| Patient8 | 1.958406154 | 0 | Patient24 | 6.028036108 | 0 |
| Patient9 | 12.06637328 | 1 | Patient25 | 3.263065959 | 0 |
| Patient10 | 3.811217638 | 0 | Patient26 | 8.361058637 | 1 |
| Patient11 | 13.01145848 | 1 | Patient27 | 11.52382275 | 1 |
| Patient12 | 8.574860311 | 0 | Patient28 | 55.8001779 | 1 |
| Patient13 | 18.87108293 | 1 | Patient29 | 23.07505879 | 1 |
| Patient14 | 8.24703894 | 1 | Patient30 | 3.547432297 | 0 |
| Patient15 | 14.39954486 | 0 | Patient31 | 7.34205363 | 1 |
| Patient16 | 23.42375798 | 1 | Patient32 | 13.28548573 | 1 |

*Radscore calculation formula:

-0.4662*wavelet-LHL_glrlm_ShortRunEmphasis+0.28125*wavelet-HLH_glszm_SizeZoneNonUniformity+0.00306*wavelet-HLH_glszm_SmallAreaLowGrayLevelEmphasis
