# Supplementary material for: Targeted sequencing of DNA/RNA combined with radiomics predicts lymph node metastasis of papillary thyroid carcinoma
Source: Cancer Imaging. 2024 Jun 17;24:75. doi: 10.1186/s40644-024-00719-2 (PMC11181663; doi:10.1186/s40644-024-00719-2)
Supplement: Supplementary file 3 — Supplementary Material 3 [file 40644_2024_719_MOESM3_ESM.doc]

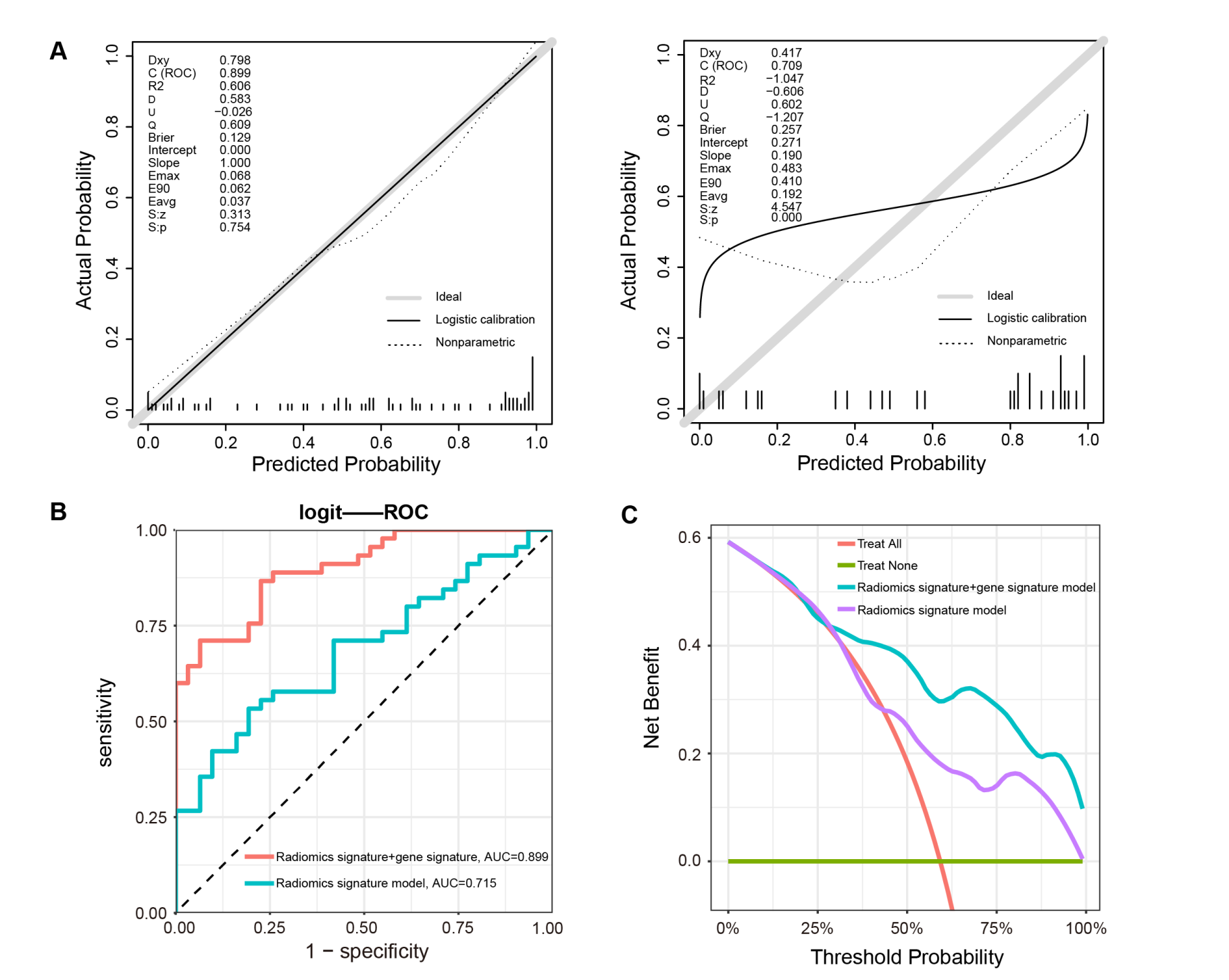

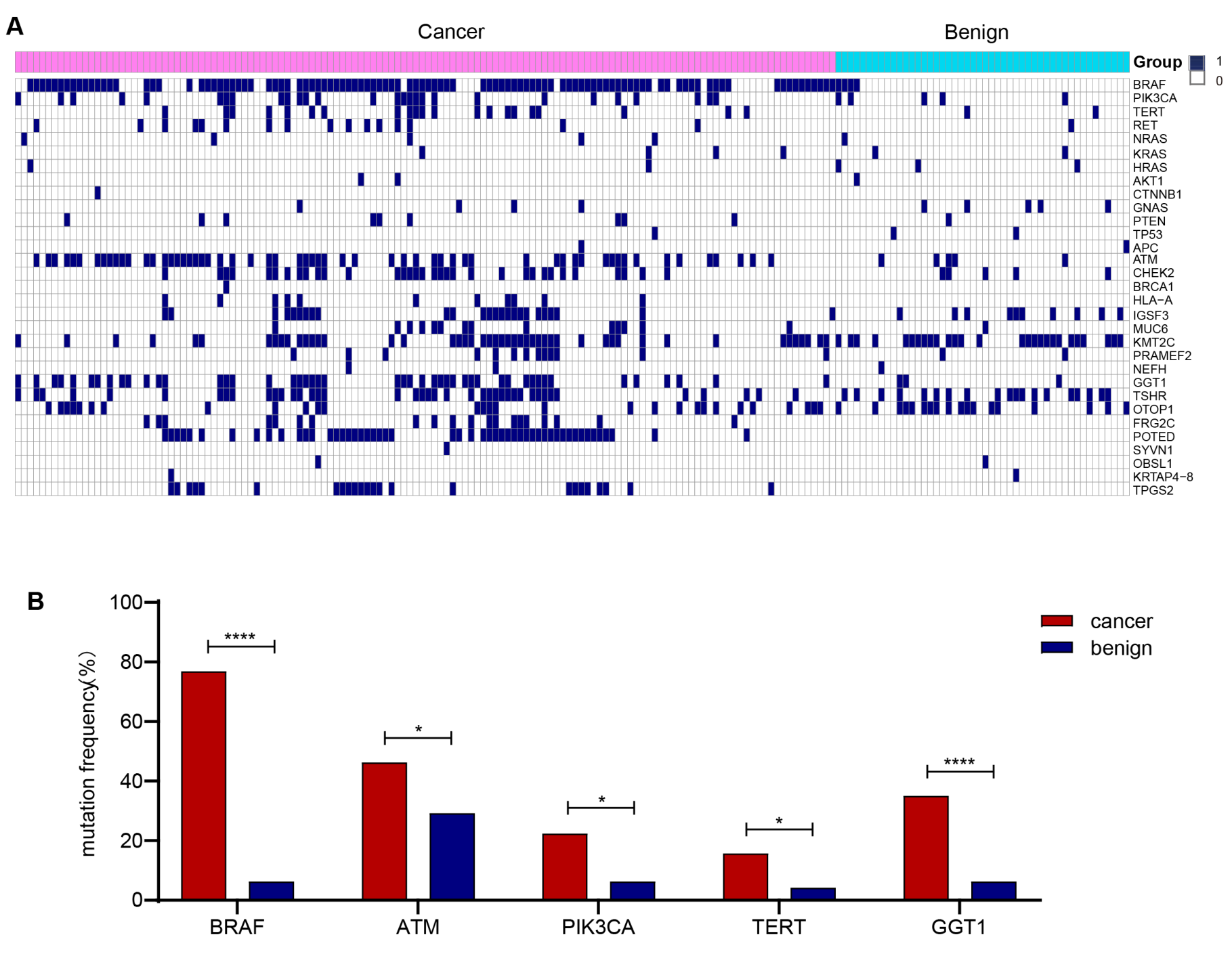

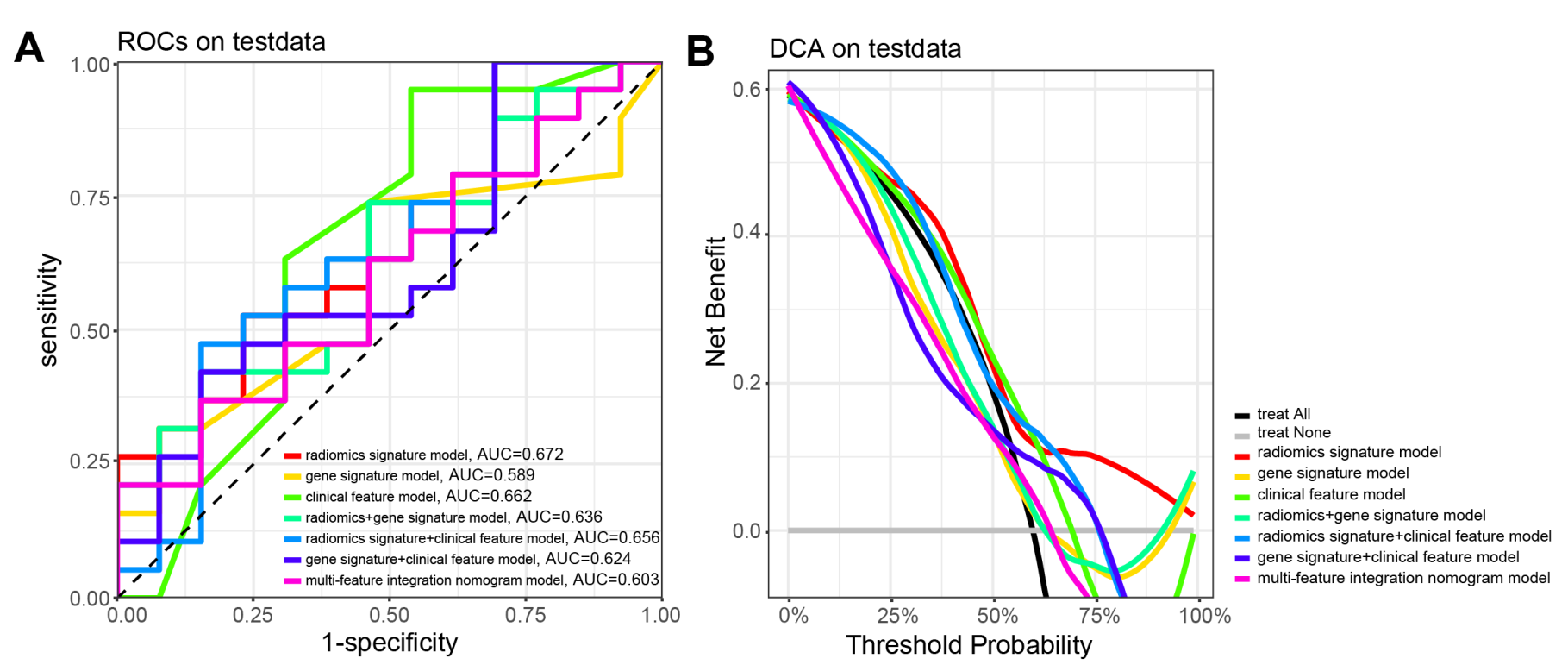

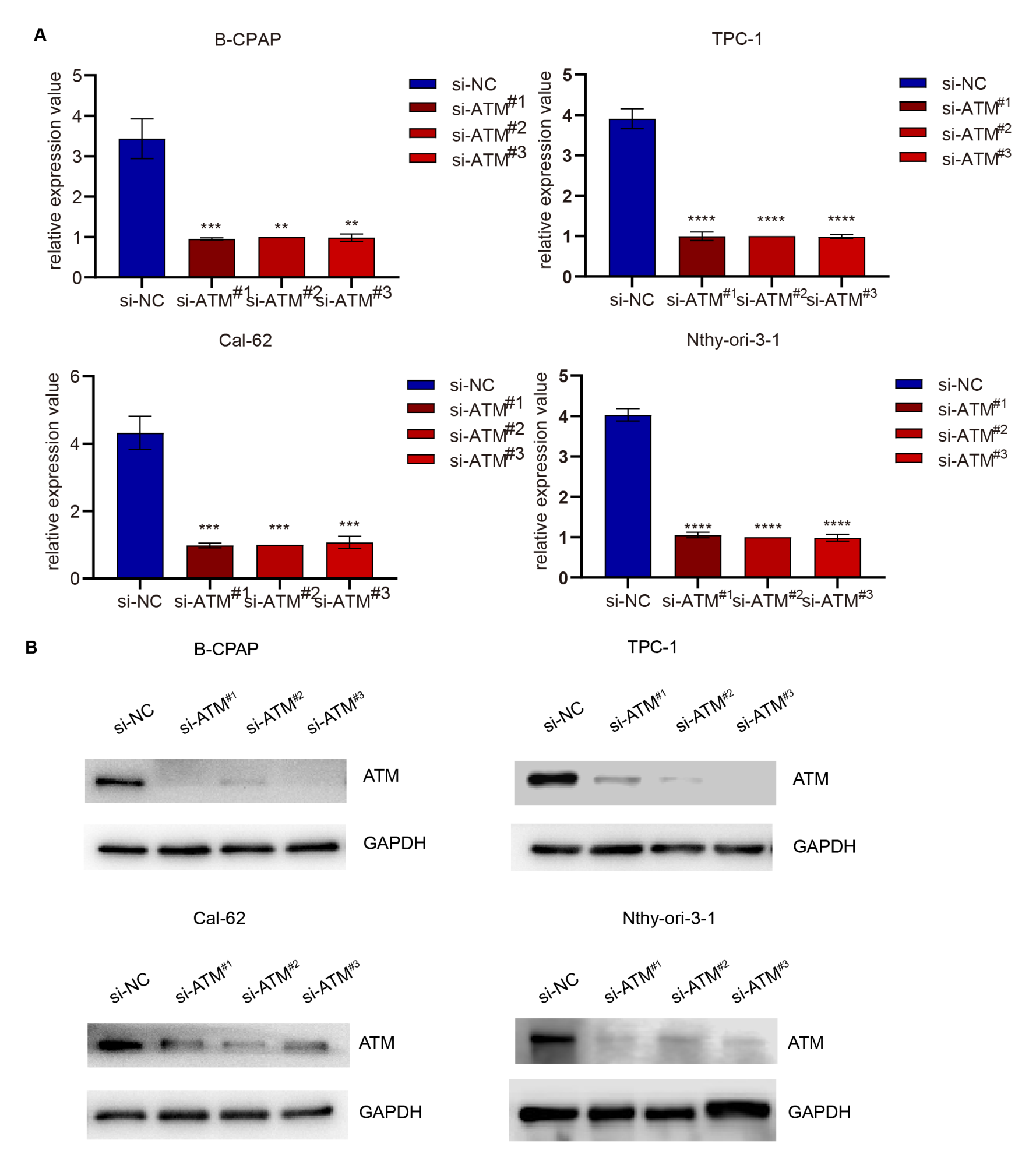

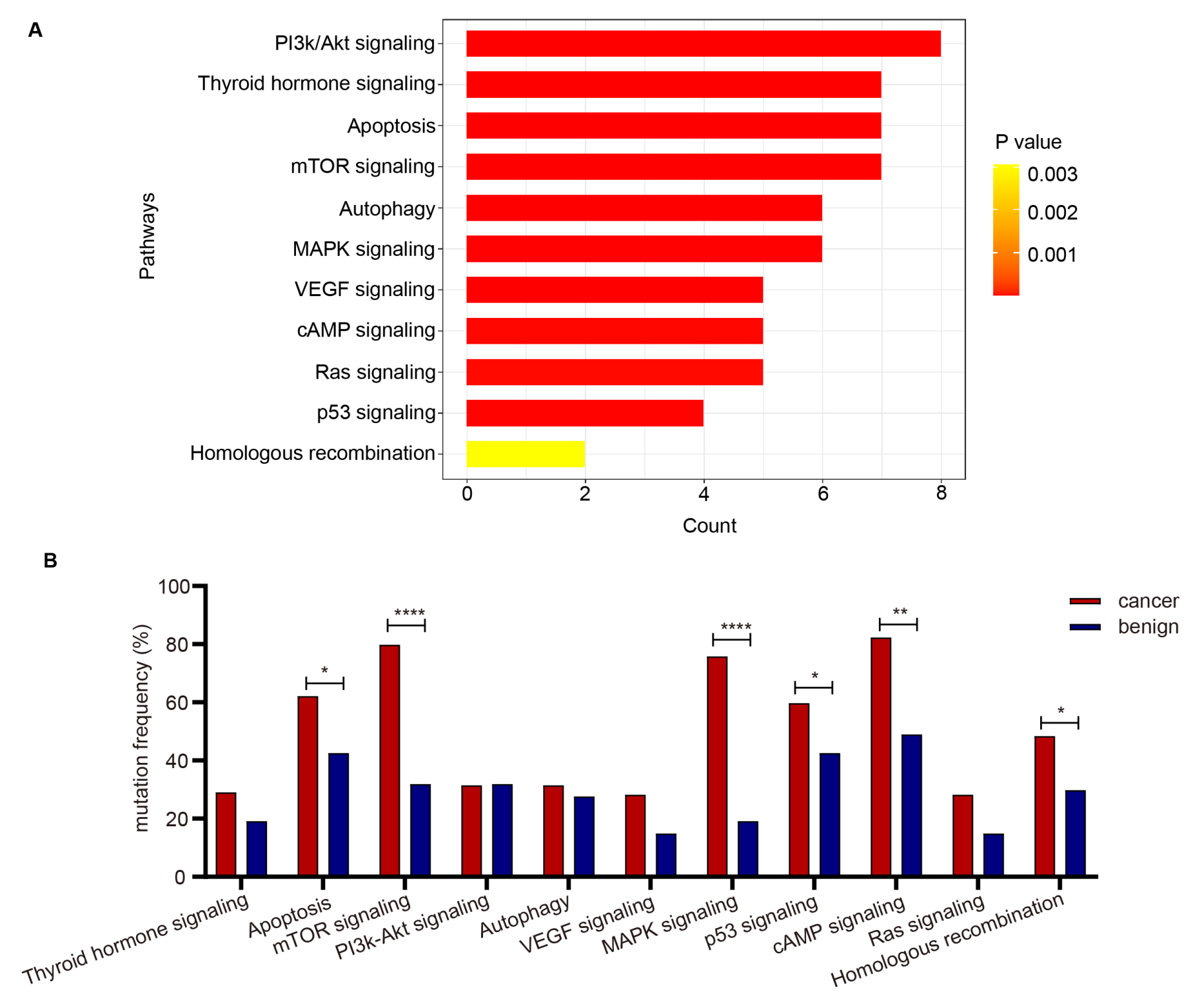

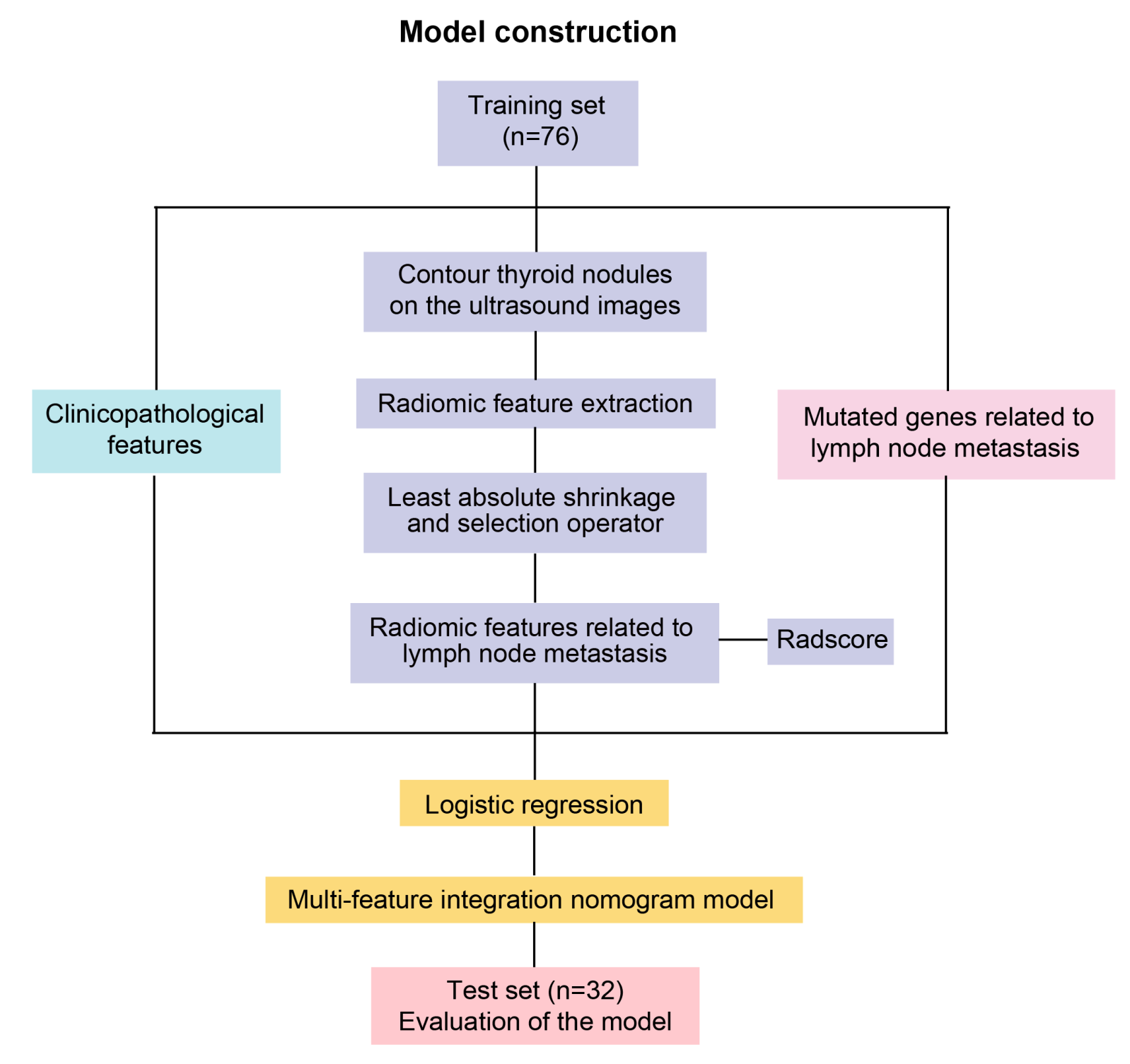

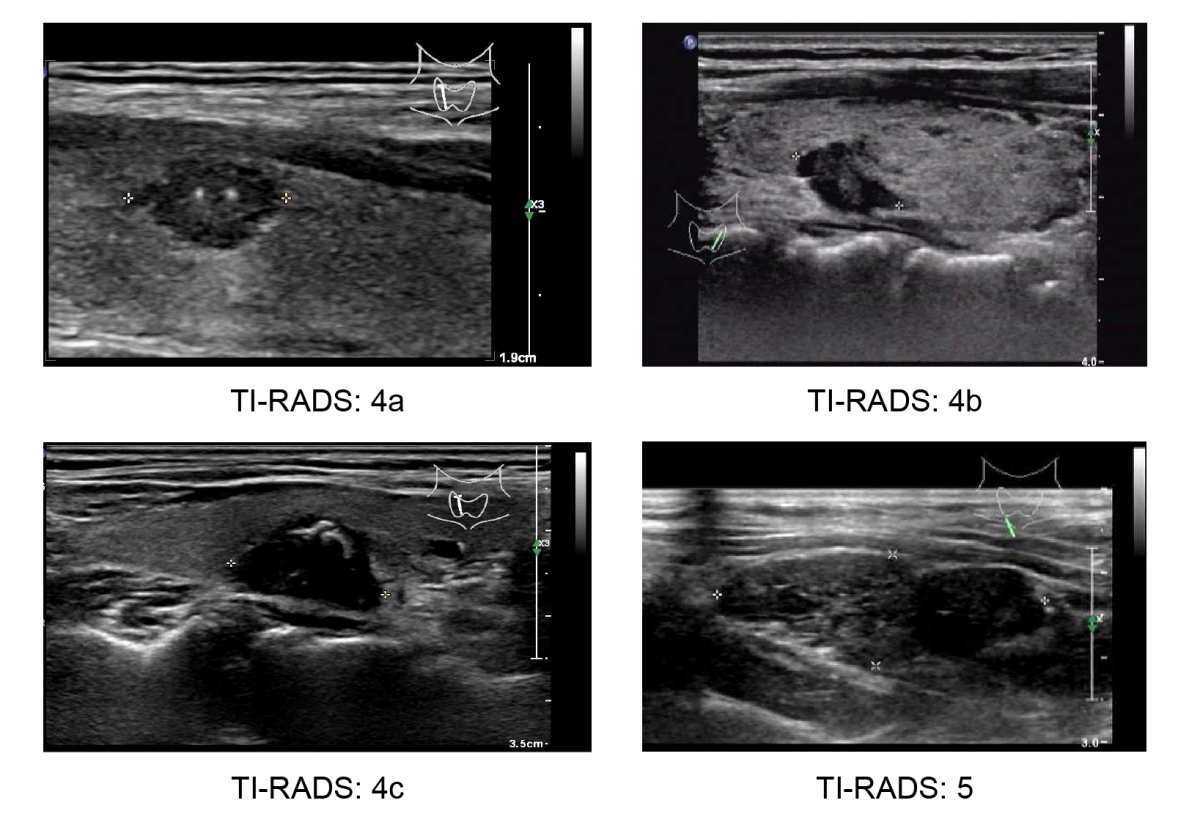


| **Table S1** Clinical pathological features of patients with PTC | |
| --- | --- |
| Clinical Pathological Parameters | Number of Patients |
| Gender | |
| Male | 30 |
| Female | 94 |
| Age | |
| ＜55 | 94 |
| ≥55 | 30 |
| Ethnicity |  |
| Chinese | 124 |
| Family history | |
| Yes | 7 |
| No | 117 |
| Tumor size | |
| ≤1cm | 63 |
| ＞1cm | 61 |
| Tumor location | |
| Left | 75 |
| Right | 47 |
| Isthmus | 2 |
| Tumor staging | |
| Ⅰ | 118 |
| Ⅱ | 2 |
| Ⅲ | 4 |
| TI-RADS | |
| 4a | 21 |
| 4b | 38 |
| 4c | 37 |
| 5 | 25 |
| 6 | 3 |
| PTC: Papillary thyroid carcinoma |  |

| **Table S2** Clinical pathological features of patients with benign thyroid nodules | |
| --- | --- |
| Clinical Pathological Parameters | Number of Patients |
| Gender | |
| Male | 14 |
| Female | 44 |
| Age | |
| ＜55 | 43 |
| ≥55 | 15 |
| Ethnicity |  |
| Chinese | 58 |
| Tumor location | |
| Left | 31 |
| Right | 27 |
| TI-RADS | |
| 2 | 1 |
| 3 | 5 |
| 4a | 43 |
| 4b | 8 |
| 4c | 1 |

| **Table S3** Detected genes in the panel | | | |
| --- | --- | --- | --- |
| Mutation | Driver oncogene | Gain of function | BRAF, RET, CTNNB1, IDH1, NRAS, HRAS, KRAS, AKT1, PIK3CA, TERT, GNAS, EGFR, EIF1AX |
|  |  | Loss of function | TP53, PTEN, APC, CDC27, ZNF717, SPOP, ZNF148 |
|  | Non-driver oncogene | DNA damage repair | CHEK2, ATM, BRCA1, OTUD4 |
|  |  | Immune | HLA-A, MUC6, IGSF3 |
|  |  | Chromosome modifier | KMT2C, PRAMEF2, NEFH, KMT2A |
|  |  | Membrane transport | TAS2R31, OBSL1, SYVN1, MUC3A |
|  |  | Hormone | TSHR |
|  |  | Metabolism | GGT1, ACHE |
|  |  | Keratin | KRTAP9-1, KRTAP4-8 |
|  |  | Ankyrin | POTED, ANKRD36 |
|  |  | Others | OTOP1, FRG2C, TPGS2, COMP, FRG1, KIAA0430, EZH1 |
| Fusion | | CCDC6-RET(3), CRTC1-MAML2, EML4-ALK(2), GOLGA5-RET, ETV6-NTRK3(2), HOOK3-RET, KTN1-RET, ERC1-RET, NCOA4-RET(4), AKAP9-BRAF, PAX8-PPARG(4), PCM1-RET, TGF-NTRK1, TPM3-NTRK1, KIF5B-RET(4), PRKAR1A-RET, STRN-ALK, TRIM24-RET(2), TRIM27-RET, TRIM33-RET, MACF1-BRAF, THADA-IGF2BP3 | |

**Table S4** The primer sequences of ACTB and ATM genes

| Genes |  |  | Primer sequences |
| --- | --- | --- | --- |
| ACTB |  |  | Forward: TGGCACCCAGCACA |
|  |  |  | Reverse: CTAAGTCATAGTCCGCCTA |
| ATM |  |  | Forward: TTGCCAGACAGCCGTGACTTAC |
|  |  |  | Reverse: AACCTCCACCTGCTCATACACAAG |

**Table S5** The mutation sites of germline variations

| Genes | Mutation sites |
| --- | --- |
| HLA-A | c. G763A :p. V255M |
| KMT2C | c. A2591G :p. E864G |
| RET | c. G2071A :p. G691S |
| POTED | c. G337A :p. G113S |
| FRG2C | c. G25A :p. D9N |
| OBSL1 | c. G3649A :p. E1217K |
| OTOP1 | c. 676_680delCTCAA :p. L226fs |
| PRAMEF2 | c. G322T :p. V108F |
| TPGS2 | c. 16_17delTC :p. S6fs |
| NEFH | c. A1958C :p. K653T |
| IGSF3 | c. A635G :p. Q212R |
|  | c. G760A :p. D254N |
| TSHR | c. G1349A :p. R450H |
| GNAS | c. C1055A :p. Pro352H |
| CHEK2 | c. G1567A :p. A523T |
| MUC6 | c. C5666T :p. T1889I |
